# Human cytomegalovirus infection triggers a paracrine senescence loop in renal epithelial cells

**DOI:** 10.1038/s42003-024-05957-5

**Published:** 2024-03-08

**Authors:** Stefano Raviola, Gloria Griffante, Andrea Iannucci, Shikha Chandel, Irene Lo Cigno, Davide Lacarbonara, Valeria Caneparo, Selina Pasquero, Francesco Favero, Davide Corà, Elena Trisolini, Renzo Boldorini, Vincenzo Cantaluppi, Santo Landolfo, Marisa Gariglio, Marco De Andrea

**Affiliations:** 1 Intrinsic Immunity Unit, CAAD - Center for Translational Research on Autoimmune and Allergic Disease, University of Eastern Piedmont, Novara, Italy; 2 Molecular Virology Unit, Department of Translational Medicine, University of Eastern Piedmont, Novara, Italy; 3 Viral Pathogenesis Unit, Department of Public Health and Pediatric Sciences, University of Turin, Medical School, Turin, Italy; 4 Bioinformatics Unit, CAAD - Center for Translational Research on Autoimmune and Allergic Disease, University of Eastern Piedmont, Novara, Italy; 5 Bioinformatics Unit, Department of Translational Medicine, University of Eastern Piedmont, Novara, Italy; 6 Pathology Unit, Department of Health Sciences, University of Eastern Piedmont, Novara, Italy; 7 Nephrology and Kidney Transplantation Unit, Department of Translational Medicine, University of Eastern Piedmont, Novara, Italy; 8 Present Address: Department of Biomedicine and Prevention, University of Rome “Tor Vergata”, Rome, Italy

**Keywords:** Viral infection, Virus-host interactions

## Abstract

Human cytomegalovirus (HCMV) is an opportunistic pathogen causing severe diseases in immunosuppressed individuals. To replicate its double-stranded DNA genome, HCMV induces profound changes in cellular homeostasis that may resemble senescence. However, it remains to be determined whether HCMV-induced senescence contributes to organ-specific pathogenesis. Here, we show a direct cytopathic effect of HCMV on primary renal proximal tubular epithelial cells (RPTECs), a natural setting of HCMV disease. We find that RPTECs are fully permissive for HCMV replication, which endows them with an inflammatory gene signature resembling the senescence-associated secretory phenotype (SASP), as confirmed by the presence of the recently established SenMayo gene set, which is not observed in retina-derived epithelial (ARPE-19) cells. Although HCMV-induced senescence is not cell-type specific, as it can be observed in both RPTECs and human fibroblasts (HFFs), only infected RPTECs show downregulation of *LAMINB1* and *KI67* mRNAs, and enhanced secretion of IL-6 and IL-8, which are well-established hallmarks of senescence. Finally, HCMV-infected RPTECs have the ability to trigger a senescence/inflammatory loop in an IL-6-dependent manner, leading to the development of a similar senescence/inflammatory phenotype in neighboring uninfected cells. Overall, our findings raise the intriguing possibility that this unique inflammatory loop contributes to HCMV-related pathogenesis in the kidney.

## Abstract (web summary)

HCMV induces a senescence-associated secretory phenotype (SASP)-like inflammatory response in renal but not retinal cells, potentially driving kidney pathogenesis through an IL-6-dependent senescence loop in neighboring uninfected cells.

## Introduction

Human cytomegalovirus (HCMV) is a widely prevalent opportunistic pathogen^1^. Generally, seroconversion of HCMV occurs in early childhood, often leading to a lifelong infection that can persist asymptomatically in most individuals. With the sole exception of congenital HCMV infection, which is the primary cause of sensorineural hearing loss and neurodevelopmental delay in children^2^, HCMV-associated diseases mainly affect immunocompromised individuals, such as transplant recipients and AIDS patients^3,4^. Thus, controlling HCMV infection/reactivation in these patients is critical for limiting its detrimental pathological consequences, such as allograft rejection and organ-specific allograft injury in kidney transplant recipients (KTRs)^5^.

HCMV belongs to a subgroup of betaherpesviruses characterized by pronounced species specificity, broad cell tropism, and prolonged viral replication cycle, which can last for up to several days^6,7^. Due to its slow-replication rate, HCMV requires a stable cell cycle environment that is achieved upon virus-mediated cell-cycle arrest at the G_1_/S border, during which cells are metabolically active and committed to DNA synthesis, prepped to support viral replication^8,9^. In order to obtain this suitable cellular environment, HCMV activates a mixture of cell-cycle stimulating and inhibiting pathways whose impact on cell fate can be cell-type-specific^10,11^. In addition, HCMV infection is known to induce replication stress, trigger host DNA damage response (DDR), and promote chromosomal instability in both permissive and non-permissive cells^12–14^. These stress response genes can transcriptionally activate the viral major immediate early enhancer promoter (MIEP), thereby inducing a stimulatory loop for immediate early gene activation that favors viral replication^15^. Consequently, infected cells may react to virus-induced genomic instability by triggering cell cycle arrest through a variety of mechanisms that can be influenced by the intrinsic permissiveness of the different cell types and potentially resulting in distinct cell fates^16–18^.

Cellular senescence is a physiological phenomenon that can occur prematurely in response to stress stimuli, including DNA damage, aging, or infections, leading to cell cycle arrest and protection from uncontrolled replication of damaged/injured cells^19^. Unlike apoptosis, which eliminates damaged cells, senescence is a complex and heterogeneous cellular program that results in stable cell growth arrest while allowing affected cells to remain viable and metabolically active^20^. Senescent cells display different phenotypic traits, including, among others, increased expression of the cyclin-dependent kinase inhibitor p16^INK4A^ and decreased expression of the major nuclear lamina component, lamin B1 (*LMNB1*), along with significant changes in protein secretion, collectively known as the senescence-associated secretory phenotype (SASP)^21^. When present in a transitory state, senescent cells can exert beneficial effects on tissue homeostasis, promoting wound healing and counteracting tissue fibrosis, whereas their aberrant accumulation and amplification through paracrine loops are associated with organ-specific disease progression or deterioration^22–24^.

More recent studies have shown how viral infections may also trigger a senescence response, generally defined as virus-induced senescence (VIS)^25–28^. In previous research, we demonstrated that HCMV infection can trigger cell senescence of human fibroblasts in a p16^INK4a^-dependent manner^29,30^. Even though it is well known that HCMV-induced pathogenesis in vivo primarily involves epithelial cells, most studies on the biology of HCMV and its host interactions have been performed in fibroblasts rather than ocular, oral, or kidney epithelial cells^31–34^. Therefore, here we sought to determine whether HCMV infection of human renal epithelial cells would promote cytopathic changes conducive to senescence, viral spread, and organ damage.

Our findings demonstrate that HCMV infection of renal proximal tubular epithelial cells (RPTECs), which are naturally susceptible to infection and associated with virus-related disease in vivo, elicits a senescence-like program characterized by stable growth arrest and secretion of senescence-associated inflammatory cytokines. Intriguingly, this senescence phenotype is passed on in an IL-6-dependent manner to neighboring uninfected kidney tubular cells indicating a paracrine mechanism of disease amplification. Notably, this paracrine loop of disease amplification is not observed in fibroblasts, suggesting its potential relevance in specific settings, such as the kidney.

## Results

### HCMV induces a robust antiviral pro-inflammatory response in fully supportive RPTECs

**Fig. 1:**
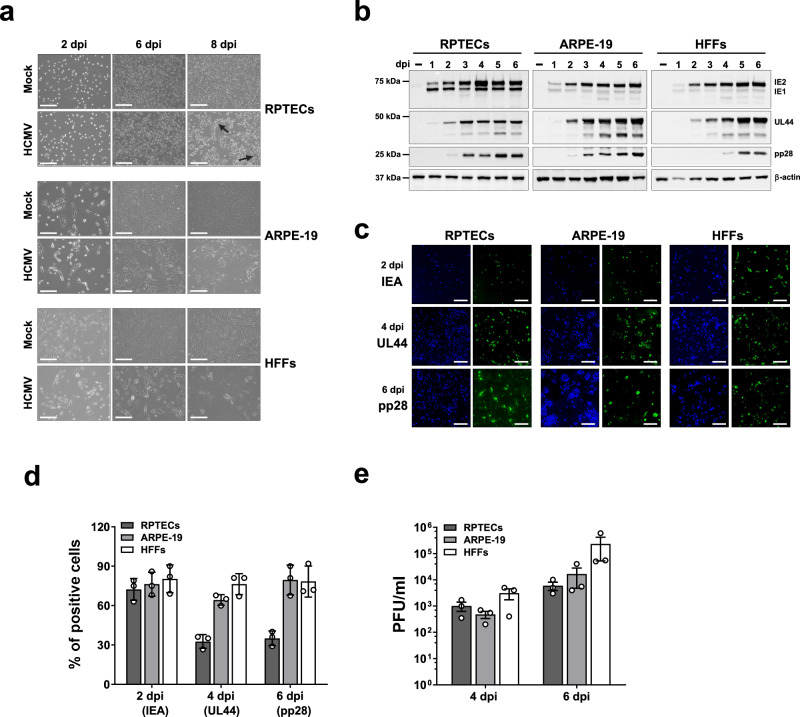
Outcome comparison of HCMV infection in different cell lines. **a** Immortalized renal proximal tubular cells (RPTECs) were infected with the HCMV TR strain (MOI of 1 PFU/cell) and observed for up to eight days post infection (8 dpi) by phase contrast micrographs. The black arrows indicate syncytia formation. HFFs and ARPE-19 cells were also included as positive controls and infected with the HCMV TR strain at MOI 0.5 and 3, respectively. Mock cells were used as negative controls. Scale bars, 250 μm. **b** Comparison of viral protein expression in RPTECs, ARPE-19 and HFFs upon HCMV infection. Protein lysates from RPTECs, ARPE-19 and HFFs infected as in (**a**) and harvested at different days post infection were subjected to immunoblotting using antibodies recognizing immediate early (IEA: IE1 and IE2), early (UL44), or late (pp28) viral antigens or anti-β-actin to show equal loading. **c** Representative indirect immunofluorescence on paraformaldehyde-fixed RPTECs, ARPE-19 and HFFs infected with HCMV for the indicated time points and stained with primary antibodies against IEA, UL44, and pp28 (green fluorescence). Cell nuclei were visualized by DAPI (blue). Scale bars, 200 μm. **d** The percentage of infected cells displaying expression of IEA (at 2 dpi), UL44 (4 dpi) and pp28 (6 dpi) was determined for all cell lines. The values were normalized to the total number of DAPI-positive cells. Image analysis was conducted using LAS X software (Leica Microsystems) and values are expressed as mean ± SD (error bars) relative to three different experiments, one of which is shown in C. **e** The production of viral particles in RPTECs, ARPE-19, and HFFs infected with HCMV TR as in (**a**) was measured by plaque assay on HFFs. At 4 and 6 dpi, viral plaques were microscopically counted and expressed as PFU/mL. Values are expressed as mean values ± SD (error bars) from three independent experiments.

To investigate the impact of HCMV infection on kidney epithelial cells, RPTECs—of note, these cells are hTERT-immortalized cells retaining normal morphological and functional properties with no genomic instability^35,36^—were infected with the HCMV TR strain^37^. Human foreskin fibroblasts (HFFs) and adult retinal pigment epithelial (ARPE-19) cells were included as these cells represent two well-established cellular models of HCMV infection^31,38^. As shown in Fig. 1a, HCMV-infected monolayers of HFFs and ARPE-19 cells showed the canonical cytopathic effect (CPE) as early as 2 days post infection (dpi), while RPTECs displayed enlargement and syncytia formation only between 6 and 8 dpi.

To further understand the viral life cycle in the three cell lines analyzed, we initially performed entry assays to quantify the number of genome-containing particles that penetrated the cells. No significant differences were observed among these cell lines (Supplementary Fig. 1). We next assessed the expression kinetics of a panel of HCMV genes representative of the different phases of the replication cycle, namely immediate-early IE (IE1 and IE2, defined as IEA), early (E), and late (L). Consistent with their cell morphology, RPTECs showed an expression pattern resembling that observed in HFFs and ARPE-19 cells. Specifically, the IE2 protein became detectable between 1 and 2 dpi in all three cell lines and remained at high levels up to 6 dpi. A similar trend, shifted from day 2 to 3, was observed for the early protein UL44, with overall lower expression levels in RPTECs compared to other cell lines. The late protein pp28 began to be expressed at 3 dpi in RPTECs and ARPE-19 cells and at 4 dpi in HFFs (Fig. 1b).

Viral protein expression was also analyzed by immunofluorescence (Fig. 1c,d), which revealed IEA expression at 2 dpi in approximately 72–80% of the cells in all three cell lines, while UL44 expression was found in ∼70% of HFFs and ARPE-19 cells and ∼33% of RPTECs at 4dpi. The latter finding is consistent with the lower expression levels observed in Western blotting. A similar trend was observed with the late protein pp28, which was expressed in ∼80% in HFFs and ARPE-19 cells, and ∼35% in RPTECs at 6 dpi.

To ascertain whether HCMV infection of RPTECs was indeed productive, we measured the virus yield in the supernatants from infected cells at 4 and 6 dpi (Fig. 1e). The highest viral loads were found in HFFs (~3 × 10^3^ and 2 × 10^5^ PFU/mL, at 4 and 6 dpi respectively), while they decreased in ARPE-19 cells (~5 × 10^2^ and ~1.7 × 10^4^ PFU/mL) and RPTECs (~1 × 10^3^ and ~6 × 10^3^ PFU/mL).

Taken together, these findings demonstrate that RPTECs support a complete HCMV lytic productive cycle and morphologically display the classical HCMV-induced CPE. However, they were less efficient in terms of viral production, as evidenced by the reduced number of cells expressing early and late viral proteins and the lower viral load values in the culture supernatants compared to those observed in HFFs or ARPE-19 cells.

**Fig. 2:**
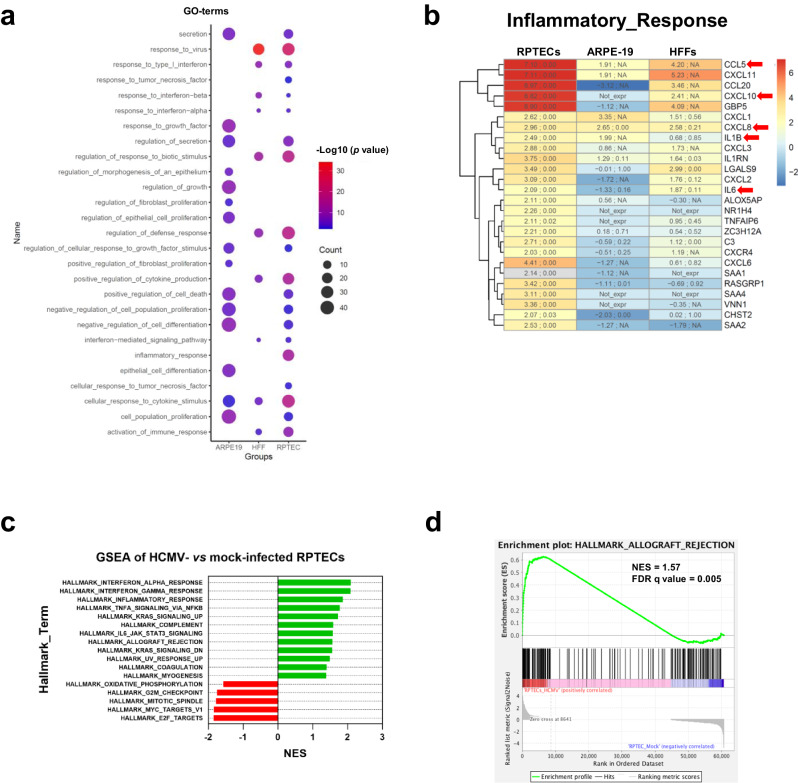
Comparison of the transcriptional response to HCMV infection in different cell lines. **a** Dot plot visualization of over-represented selected gene ontology (GO) terms in RPTECs, ARPE-19 and HFFs. Over-representation analyses were performed using Metascape, as reported in the Materials and Methods section. Each dot on the plot represents a GO term, with the color indicating the -Log10 (p value) for the enrichment, and the size representing the number of genes enriched in the gene set. **b** Heatmap showing unsupervised hierarchical clustering of DEGs enriched in the GO term “Inflammatory response” in HCMV-infected *vs*. mock-infected cells based on Metascape analysis. For each gene, log2 fold change (log2FC) and false rate discovery (FDR) between HCMV- and mock-infected cells are reported for each cell line, separated by semicolon. When TPM < 1, “*Not_expr*” string is indicated. **c** Gene set enrichment analysis (GSEA) was conducted to investigate the enrichment of hallmark gene sets from MSigDB Collection in HCMV-infected RPTECs *vs*. mock-infected controls. Positive normalized enrichment score (NES) indicate enrichment (green bars), whereas negative NES indicate downregulation in virus-infected cells (red bars). Hallmarks with a |NES | > 1 and FDR q-value < 0.05 are considered statistically significant and are shown. **d** GSEA of the comparison between HCMV-infected and mock-infected RPTECs showing the enrichment of hallmark pathways of allograft rejection. The green curve in the GSEA plot represents the enrichment score curve. Genes on the far left (red) correlate with HCMV-infected cells, whereas genes on the far right (blue) correlate with mock-infected cells. The vertical black lines indicate the position of each gene in the gene set analyzed. NES, false discover rate (FDR), and nominal *P*-value are shown for this hallmark.

To gain a deeper understanding of the transcriptional profile of RPTECs upon HCMV infection, we conducted transcriptome analysis at 2 dpi. Total RNA from two biological replicates of infected *vs* non-infected cells was subjected to high-throughput sequencing for poly-A^+^ RNAs, and their transcriptome profiles were compared to those of HCMV-infected HFFs and ARPE-19 cells available in the GEO database^39^. Functional annotation of differentially expressed genes (DEGs) between HCMV- and mock-infected cells for each cell line showed significant enrichment in several biological processes. In both HCMV-infected RPTECs and HFFs but not in ARPE-19 cells, the following biological processes were significantly enriched among the DEGs: “response to virus”, “response to type I interferon”, “regulation of response to biotic stimulus”, “regulation of defense response” and “positive regulation of cytokine production”. Conversely, HCMV-infected ARPE-19 cells showed almost equal enrichment of processes such as “response to growth factor”, “regulation of growth”, “regulation of epithelial cell proliferation”, “epithelial cell differentiation”, and “regulation of morphogenesis of an epithelium”. Interestingly, the gene ontology (GO) term “inflammatory response” was identified specifically in HCMV-infected RPTECs, but not in the other two cell lines (Fig. 2a). As shown in the heatmap in Fig. 2b, analysis of DEGs associated with the “inflammatory response” revealed high expression levels of several paradigmatic inflammatory cytokines, such as IL-6, IL-1β, CXCL8 (IL-8), CCL5 (RANTES) and CXCL10 (IP-10), in infected RPTECs. These cytokines remained unchanged upon infection in both ARPE-19 cells and HFFs. In addition, an exploratory study was conducted by gene set enrichment analysis (GSEA) using the hallmark gene set of HCMV- *vs*. mock-infected RPTECs (Fig. 2c), ARPE-19 and HFFs (Supplementary Fig. 2). This analysis revealed a significant positive enrichment of the “hallmark of allograft rejection” gene set only in RPTECs, with a positive normalized enrichment score (NES) of 1.57 and a significant false discovery rate (FDR) q-value of 0.005 (Fig. 2d).

Overall, the gene signature found in HCMV-infected RPTECs uncovers a strikingly unique inflammatory response that is not evident in the available data set from infected HFFs or ARPE-19 cells.

**Fig. 3:**
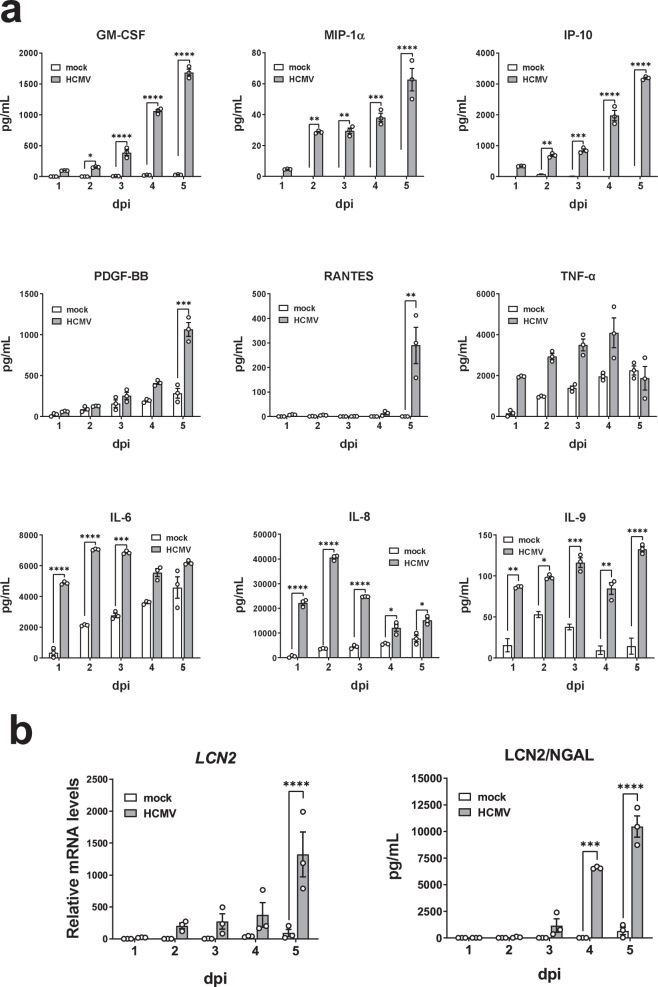
HCMV infection triggers a robust detrimental antiviral/pro-inflammatory response in RPTECs. **a** Multiplex analysis of secreted cytokines and chemokines in the supernatants from HCMV-infected RPTECs (grey bars) (MOI 1) at different time points after infection. Of the 27 cytokines and chemokines assayed in cell-free medium samples, the following 9 were found to be secreted at much higher levels compared to mock controls (white bars): granulocyte-macrophage colony-stimulating factor (GM-CSF), macrophage inflammatory protein 1-alpha (MIP-1-α/CCL3), interferon-inducible protein 10 (IP-10/CXCL10), platelet-derived growth factor-BB (PDGF-BB), regulated upon activation, normal T-cell expressed and secreted (RANTES), tumor necrosis factor-alpha (TNF-α), interleukin-6 (IL-6), interleukin-8 (IL-8), interleukin-9 (IL-9). Data are expressed as mean values ± SD from three independent experiments (*P < 0.05, **P < 0.01, ***P < 0.001, ****P < 0.0001, two-way ANOVA followed by Dunnett’s test). **b** qPCR analysis of mRNA expression of lipocalin 2 or neutrophil gelatinase-associated lipocalin (*LCN2*, left panel) or ELISA measurement of the secreted protein (LCN2/NGAL, right panel) in HCMV- (grey bars) or mock-infected RPTECs (white bars) at the indicated time points. Data are expressed as mean values ± SD from three independent experiments (***P < 0.001, ****P < 0.0001, two-way ANOVA followed by Dunnett’s test).

To gain more insight into the inflammatory signature displayed by HCMV-infected RPTECs, we compared their cytokine secretion kinetics with those of uninfected cells by Bio-Plex analysis. As shown in Fig. 3a, several inflammatory cytokines were significantly upregulated following HCMV infection, albeit with different induction kinetics. Specifically, GM-CSF, MIP1-α, and IP-10—the latter being a chemokine correlated with acute tubular injury and kidney graft rejection^40^—showed a gradual increase in secretion over time in HCMV- but not mock-infected cells, reaching peak levels at 5 dpi. In contrast, we could only detect significant levels of secreted PDGF-BB and RANTES in the supernatants from HCMV-infected cells at 5 dpi. Lastly, other pro-inflammatory cytokines, such as TNF-α, IL-6, IL-8, and IL-9, exhibited significant upregulation at 1dpi compared to controls, and with no further induction at later time points post infection.

As the inflammatory milieu triggered by HCMV infection in renal cells suggested cell damage, we investigated the expression levels of lipocalin 2 (LCN2), also known as neutrophil gelatinase-associated lipocalin or NGAL, a well-known biomarker of renal tubular injury^41^. LCN2 expression was significantly increased at both the mRNA and protein levels in HCMV- *vs*. mock-infected cells, with peak values observed at 5 dpi (Fig. 3b, right panel).

Collectively, our findings indicate that HCMV elicits robust antiviral pro-inflammatory responses in RPTECs, along with the induction of kidney damage markers.

### HCMV infection induces senescence in RPTECs, less in HFFs, but not in ARPE-19 cells

**Fig. 4:**
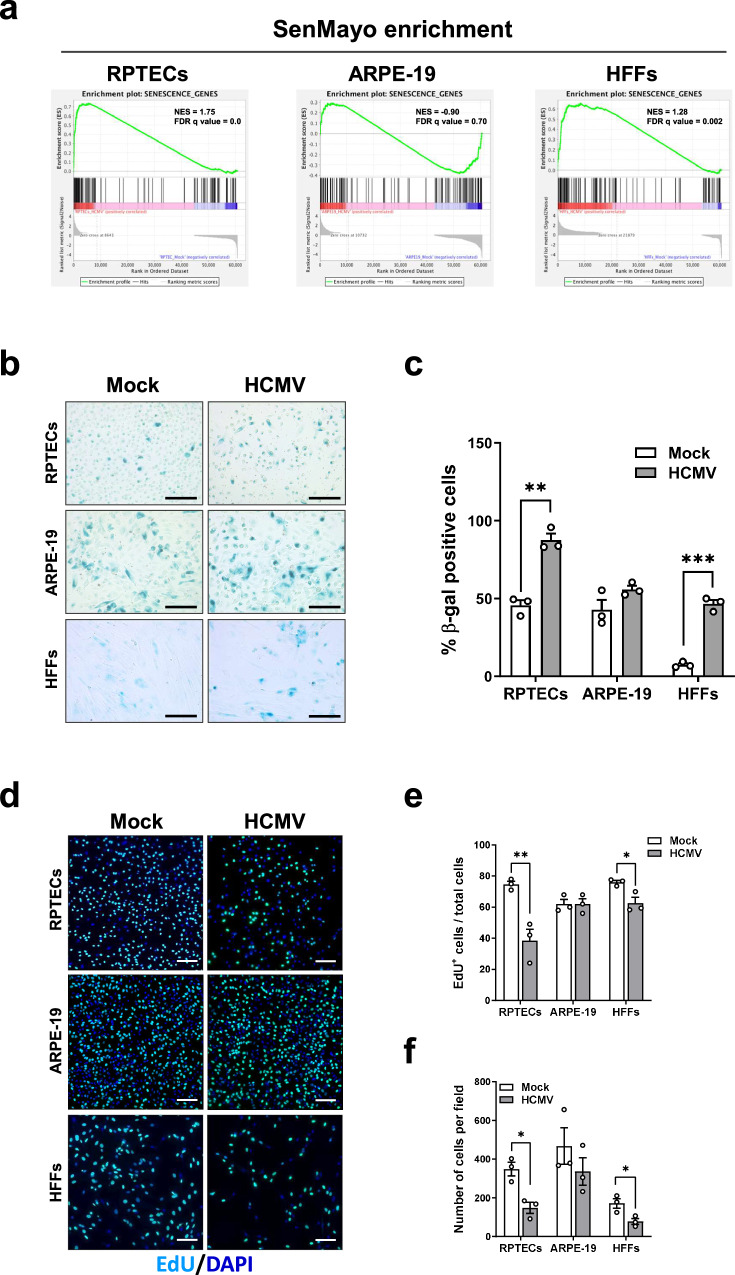
Induction of a pro-inflammatory senescence phenotype in RPTECs and HFFs upon HCMV infection. **a** GSEA of the comparison between HCMV-infected and mock-infected cells, showing the enrichment of senescence genes associated with the SenMayo signature^43^ in RPTECs and HFFs. The enrichment score curve is depicted in green, with genes on the far left (red) correlating with HCMV-infected cells, and genes on the far right (blue) correlating with mock-infected cells. The vertical black lines indicate the position of each gene in the gene set analyzed. NES and FDR are shown. **b** Representative images of senescence-associated β-gal (SA-β-gal) staining in HCMV- or mock-infected RPTECs, ARPE-19 and HFFs at 3dpi (MOI 1, 3 and 0.5, respectively). The blue areas represent positive SA-β-gal staining. Scale bars, 250 μm. **c** Histograms representing the percentage of SA-β-gal^+^ RPTECs, ARPE-19 and HFFs. Data are expressed as mean values ± SD from three independent experiments (**P < 0.01, ***P < 0.001; multiple unpaired t-test). **d** RPTECs, ARPE-19 and HFFs were infected as described in (b). Subsequently, cells were incubated with EdU for 20 h and stained as described in the Material and Methods section. DAPI was used to counterstain the nuclei. Scale bars, 200 μm. **e** Histograms representing the percentage of EdU^+^ cells. Data expressed as mean values ± SD from three independent experiments (*P < 0.05; multiple unpaired t-test). **f** The same images used in (**d**) were also used to calculate the total number of cells per field (*P < 0.05, **P < 0.01; multiple unpaired t-test).

Next, as many of the cytokines we found to be upregulated in HCMV-infected RPTECs had previously been linked to SASP^42^, we explored the possibility that HCMV infection could activate a senescence program in these cells. To assess the occurrence of a senescence signature in our model, we took advantage of the recently defined and validated SenMayo gene set^43^, which identifies senescent cells and predicts senescence-associated pathways across various tissues. As shown in Fig. 4a, when we tested SenMayo enrichment by comparing the available mRNA-seq dataset with our transcriptome analysis, we observed a significant enrichment in senescence/SASP in HCMV-infected RPTECs and HFFs (FDR q-value = 0.000 and = 0.002, respectively), while no significant enrichment was found in infected ARPE-19 (FDR q-value = 0.70). Consistent with these findings, HCMV-infected RPTECs displayed increased senescence-associated (SA-β-gal) activity at neutral pH at 3 dpi compared to mock-infected cells (~88% *vs*. ~45%; *P* < 0.01). Similar results were obtained in HCMV-infected HFFs (∼45% *vs* ∼8%; *P* < 0.001), indicating that both cell types underwent senescence (Fig. 4b,c). Furthermore, after 3 days of HCMV infection, both RPTECs and HFFs were growth arrested, as judged by decreased EdU incorporation (∼38% *vs* 75% in RPTECs, and ∼63% *vs* 76% in HFFs; *P* < 0.01 and *P* < 0.05, respectively) (Fig. 4d,e) and a reduced total cell number per field (*P* < 0.05 for both) (Fig. 4f) compared to control cells. In contrast, in ARPE-19 cells, neither the percentage of EdU-positive cells (∼60%) nor the total cell number per field (*P* > 0.05) were significantly affected by HCMV infection, and the percentage of cells displaying SA-β-gal activity did not change significantly in infected *vs*. uninfected cells (Fig. 4d–f). The results of the propidium iodide (PI) permeabilization assay, which assessed the cell death ratio after 3 days of infection, showed no significant differences, ruling out any bias due to increased cell death in infected *vs*. uninfected cells (Supplementary Fig. 3). Therefore, these observations collectively suggest that senescence upon HCMV infection occurs exclusively in RPTECs and HFFs.

**Fig. 5:**
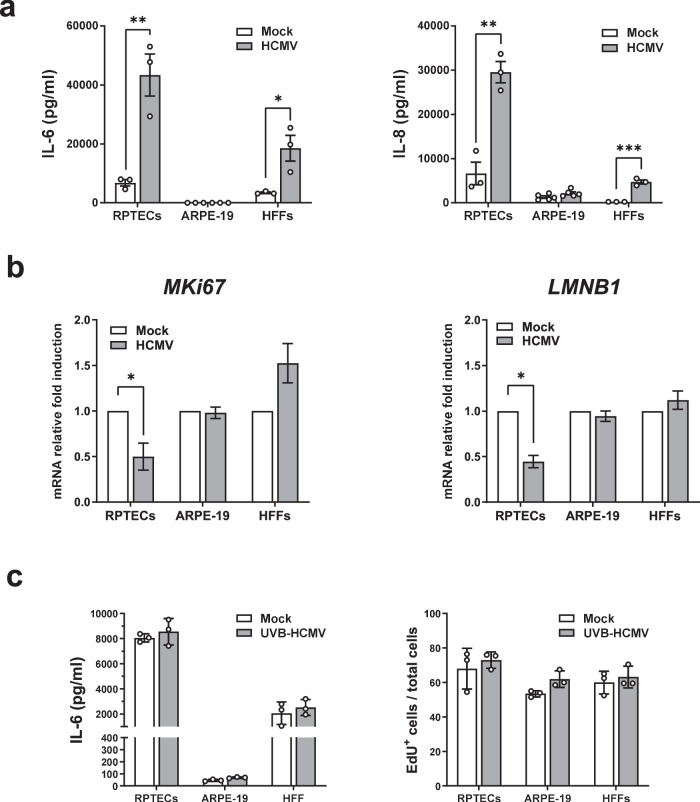
The senescence-associated inflammatory phenotype is more robust in RPTECs and HCMV replication-dependent. **a** The protein concentration of IL-6 and IL-8 was evaluated by ELISA in supernatants from HCMV- or mock-infected RPTECs, ARPE-19 and HFFs at 3dpi (MOI 1, 3 and 0.5, respectively). Data are expressed as mean values ± SD of three independent experiments (*P < 0.05, **P < 0.01, multiple unpaired t-test). **b** qPCR analysis of *KI67* and *LAMINB1* mRNA expression levels from total RNA extracts harvested at 3 dpi. Values are normalized to *18* *S* gene expression and plotted as fold induction over mock-treated cells. qRT-PCR data are presented as mean values from biological triplicates. Error bars indicate SD (*P < 0.05, multiple unpaired t-test). **c** UVB inactivated virus (UVB-HCMV) was used to evaluate the impact of HCMV replication on the release of IL-6 by ELISA (left panel) or on EdU incorporation (right panel) in RPTECs, ARPE-19 and HFFs. Data are expressed as mean values ± SD from three independent experiments.

We next proceeded to characterize the effects of HCMV infection on the secretion of IL-6 and IL-8, two inflammatory cytokines often associated with a senescent phenotype. As shown in Fig. 5a, HCMV-infected RPTECs and HFFs released higher amounts of IL-6 and IL-8 into their culture supernatants compared to mock-infected cells (IL-6: ∼6-fold and ∼8-fold in RPTECs and HFFs, respectively; IL-8: ∼4-fold and ∼8-fold in RPTECs and HFFs, respectively). Notably, the absolute amount of IL-6 and IL-8 found in the culture supernatants was significantly higher in HCMV-infected RPTECs than in similarly infected HFFs (*P* < 0.001 and *P* < 0.05, respectively), while their secretion was negligible in ARPE-19 cells. Surprisingly, HCMV infection downregulated the mRNA expression levels of the proliferation marker Ki67 (*MKi67*) exclusively in RPTECs (*P* < 0.01) but not in HFFs or ARPE-19 cells (Fig. 5b, left panel). Likewise, the mRNA expression levels of lamin B1 (*LMNB1*), a protein involved in global and local chromatin changes and whose downregulation is considered a reliable biomarker for various types of senescence^44,45^, were significantly reduced in HCMV-infected RPTECs (*P* < 0.05) (Fig. 5b, right panel), whereas they remained largely unaltered in both HFFs and ARPE-19 cells.

To rule out that the observed IL-6 induction upon HCMV infection was a consequence of the virus entry process and independent from viral replication, we infected the cells with UVB-inactivated HCMV, confirmed to be inactive by assessing its ability to produce viral immediate early antigen (IEA) compared to intact virus Supplementary Fig. 4. As shown in Fig. 5c (left panel), UVB-inactivated HCMV infection failed to stimulate IL-6 production in all three cell lines, confirming that its induction requires active replication. Similarly, EdU incorporation was unaffected in all three cell lines upon UVB-inactivated HCMV infection (Fig. 5c, right panel). These findings suggest that senescence induction upon HCMV infection is highly dependent on cell type. It is present in RPTECs and HFFs but absent in ARPE-19 cells.

It is also important to point out that the senescence-associated inflammatory phenotype exhibited by RPTECs seems to be more pronounced than that observed in HFFs. This observation gains further significance in light of our findings from tissue sections of a preterm child with a full-blown HCMV infection (refer to the last paragraph and Supplementary Fig. 5). In these sections, numerous HCMV-infected renal tubular cells showed massive cytoplasmic accumulations of hyper-pigmented lipofuscin (LF) granules, a widely recognized in vivo hallmark of senescence. This parallel between the pronounced phenotype in RPTECs and the in vivo evidence from HCMV-infected tissue strengthens the relevance of our observations.

### An IL-6 dependent senescence paracrine loop is induced in infected-RPTECs but not in infected-HFFs or ARPE-19 cells

**Fig. 6:**
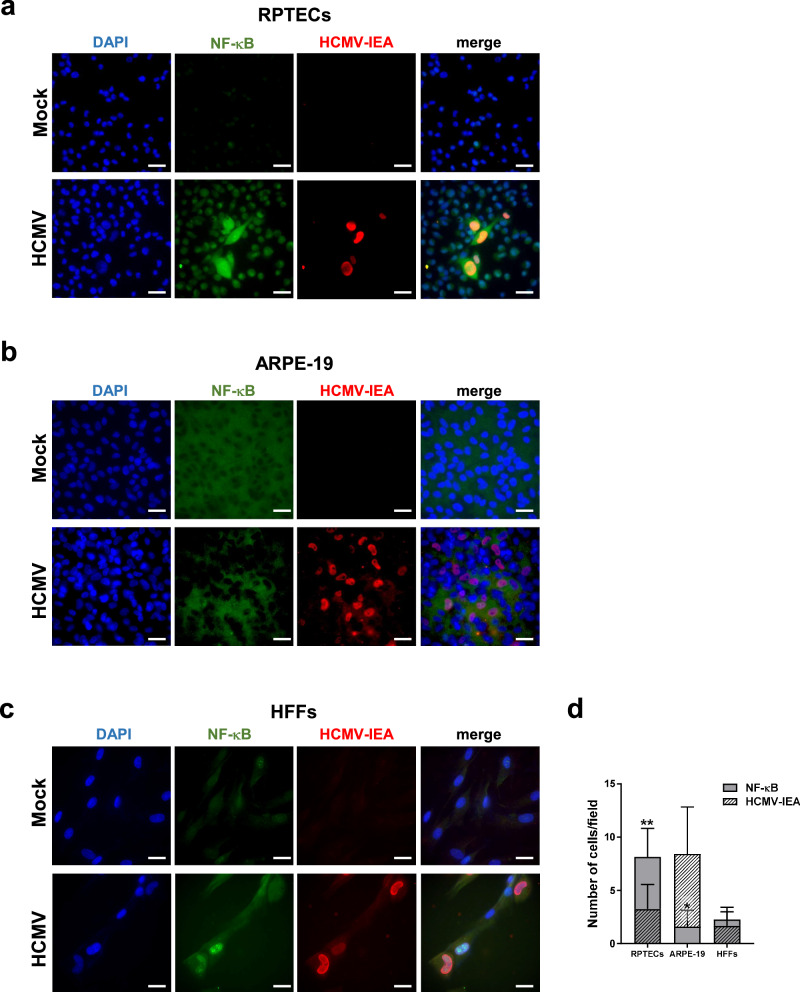
HCMV infection triggers NF-κB nuclear translocation in bystander uninfected RPTECs but not HFFs or ARPE-19 cells. Representative immunofluorescence co-staining images showing NF-κB (in green) and HCMV immediate early antigen (IEA, in red) in mock- or HCMV-infected RPTECs (**a**), ARPE-19 (**b**) and HFFs (**c**) at 3dpi. Cell nuclei are visualized through DAPI staining (blue). Scale bars, 30 μm. **d** Histograms representing the number of NF-κB-positive nuclei and IEA-positive cells per field in HCMV-infected cells. Three random fields of view per cell line from 3 different experimental replicates (at ×63 magnification) were captured, and the cell counts were graphed (*P < 0.05, **P < 0.01, multiple unpaired t-test).

To better characterize the senescence profile of HCMV-infected cells and determine the fate of infected *vs* neighboring uninfected cells, we performed co-staining of the three cell lines for a panel of senescence/inflammatory markers along with a marker of viral infection. Immunofluorescence analysis revealed NF-κB nuclear translocation in both RPTECs and HFFs, which coincided with the expression of viral IEA (Fig. 6a,c). Intriguingly, we observed that a significant proportion of NF-κB-positive nuclei of infected RPTECs but not HFFs did not express IEA (*P* < 0.01; Fig. 6d), suggesting a possible spread of pro-inflammatory signals to neighboring uninfected cells. In contrast, in ARPE-19 cells the proportion of IEA-positive nuclei was much higher and exhibited limited co-staining with anti-NF-κB antibodies, consistent with the reduced inflammatory reaction elicited by HCMV infection in these cells.

**Fig. 7:**
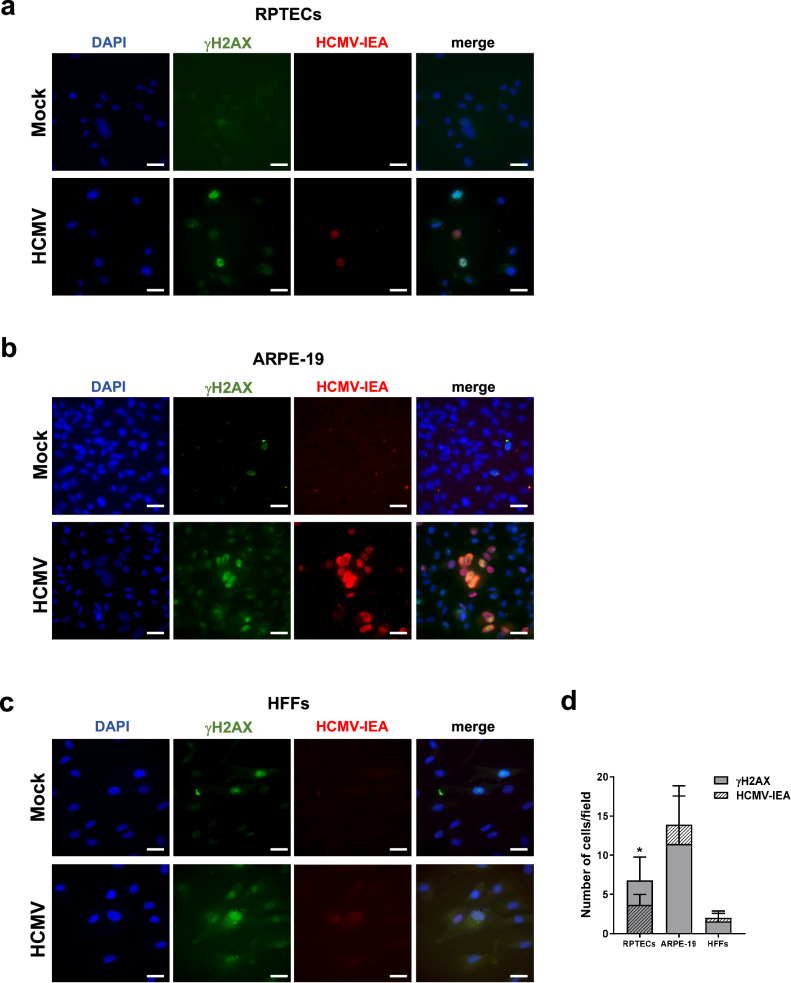
HCMV infection triggers the expression of the phosphorylated form of histone H2AX (γH2AX) in bystander uninfected RPTECs but not HFFs or ARPE-19. Representative immunofluorescence co-staining for γH2AX (in green) and HCMV immediate early antigen (IEA, in red) in mock- or HCMV-infected RPTECs (**a**), ARPE-19 (**b**) and HFFs (**c**) at 3dpi. Cell nuclei are visualized by DAPI staining (blue). Scale bars, 30 μm. **d** Histograms represent the number of γH2AX-positive nuclei and IEA positive cells per field in HCMV-infected cells. Three random fields of view per cell line from 3 different experimental replicates (at ×63 magnification) were captured, and the cell counts were graphed (*P < 0.05, multiple unpaired t-test).

**Fig. 8:**
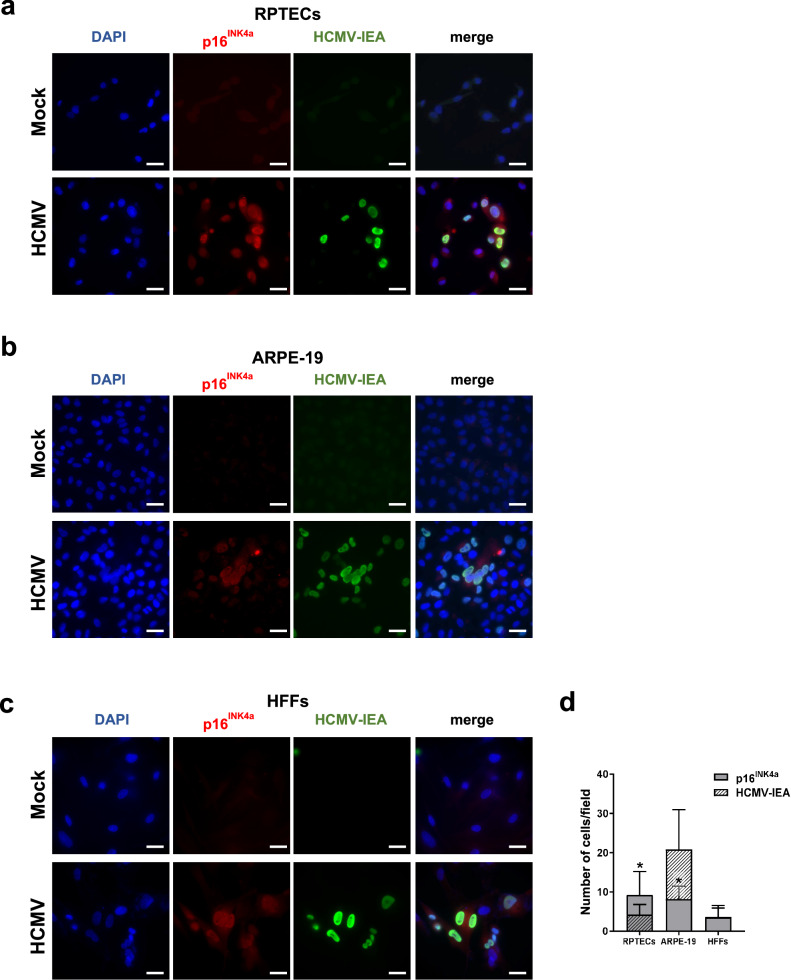
HCMV infection triggers the expression of p16^INK4a^ in bystander uninfected RPTECs but not HFFs or ARPE-19. Representative immunofluorescence staining for p16^INK4a^ (in red) and HCMV immediate early antigen (IEA, in green) in mock- or HCMV-infected RPTECs (**a**), ARPE-19 (**b**) or HFFs (**c**). Cell nuclei were visualized by DAPI (blue). Scale bars, 30 μm. **d** Histograms representing the number of p16^INK4a^ and IEA positive cells per field in HCMV-infected cells. Three random fields of view per cell line from 3 different experimental replicates (at ×63 magnification) were captured, and the cell counts were graphed (*P < 0.05, multiple unpaired t-test).

Given the association between senescence and persistent DNA damage, we asked whether the induction of the phosphorylated form of histone H2AX (γH2AX), a widely used marker for DNA damage and chromosomal instability^46^, would be influenced by HCMV infection. Based on the results shown in Fig. 6, we anticipated the presence of γH2AX staining also in the nuclei of bystander uninfected RPTECs but not in HFFs or ARPE-19 cells. Co-staining of HCMV-infected cells with antibodies against γH2AX and IEA revealed varying proportions of γH2AX-positive nuclei/field in the three cell lines: significantly higher in ARPE-19 cells, intermediate in RPTECs, and very low in HFFs (Fig. 7a–c). Surprisingly, only in infected RPTECs a significant proportion of γH2AX-positive cells did not display the viral marker (*P* < 0.05; Fig. 7d), confirming the spread of DNA damage signals from infected to bystander uninfected RPTECs^47^. A similar pattern was observed when the same cultures were co-stained with anti-p16^INK4a^ and anti-IEA antibody. Once again, a significant proportion of p16^INK4a^ -positive cells did not display the viral marker in RPTEC cultures (*P* < 0.05; Fig. 8a,d). Conversely, p16^INK4a^ staining was solely restricted to infected HFFs, whereas ARPE19 showed nuclear p16^INK4a^ reactivity in less than 50% of the IEA-positive cells (Fig. 8b–d). Thus, these results strongly suggest the existence of a secondary paracrine senescence program specifically in HCMV-infected RPTECs, which was not observed in the other two cell lines investigated.

**Fig. 9:**
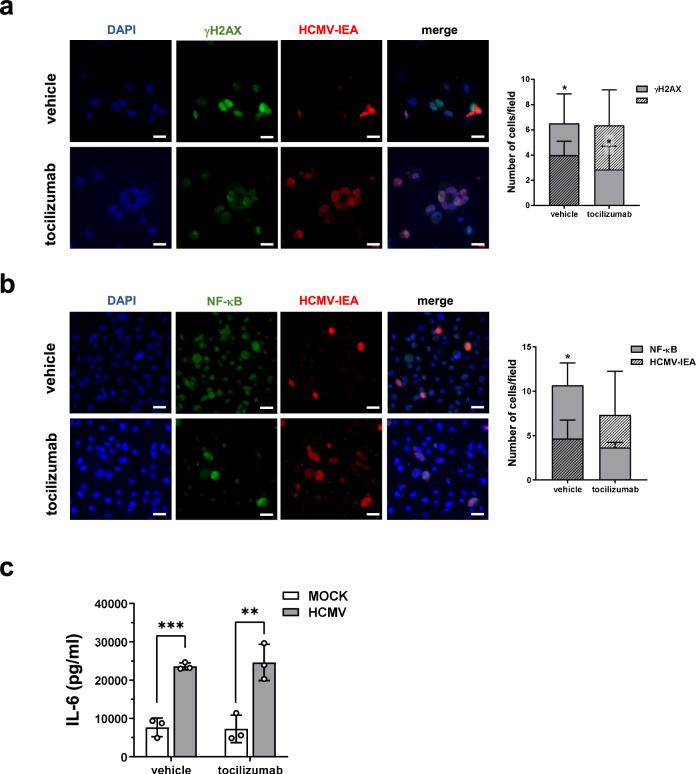
The IL-6-receptor inhibitor tocilizumab impairs the HCMV-mediated paracrine effects in RPTECs. RPTECs were infected with HCMV TR strain (MOI 1) for 3 days in the presence of IL-6R inhibitor tocilizumab (TCZ) (25 μg/mL) or vehicle control (DMSO). **a**–**b** Representative immunofluorescence images of co-stainings for γH2AX (**a**) and NF-κB (**b**) in green, and HCMV immediate early antigen (IEA, in red). Cell nuclei were visualized by DAPI (blue). Histograms on the right of each panel represent the number of γH2AX- or NF-κB-positive nuclei and IEA-positive cells per field in HCMV-infected cells. Three random fields of view per cell line from 3 different experimental replicates (at ×63 magnification) were captured and cell number graphed (*P < 0.05, multiple unpaired t-test). Scale bars, 30 μm. **c** Protein **c**oncentration of IL-6 was evaluated by ELISA in supernatants from mock- or HCMV-infected RPTECs (MOI 1) treated with TCZ or with vehicle control (DMSO). Data are expressed as mean values ± SD of three independent experiments (**P < 0.01, ***P < 0.001, multiple unpaired t-test).

Given the enrichment of the “hallmark of IL-6/JAK-STAT3 signaling” (FDR *q* value = 0.005, NES of 1.585) in HCMV- vs. mock-infected RPTECs (Fig. 2c), and considering that this pathway has been implicated in paracrine senescence induction^48^, we aimed to assess the involvement of IL-6 in our model of paracrine senescence by exposing infected cells to the IL-6R inhibitor tocilizumab (TCZ). As shown in Fig. 9a, the addition of TCZ to the cell cultures markedly reduced the proportion of γH2AX-positive cells, which was limited to about 50% of IEA-positive cells (*P* < 0.05). This result indicates that the spread of DNA damage signals observed in vehicle-treated cells was lost in the presence of TCZ. Similar results were obtained when we quantified the nuclear signal of NF-κB. In the presence of TCZ, the proportion of NF-κB-positive nuclei was reduced compared to vehicle-treated cells, and only approximately half of the IEA-positive cells displayed NF-κB staining (Fig. 9b). Importantly, TCZ did not interfere with IL-6 release in the culture supernatants upon HCMV infection (Fig. 9c), implying that this inhibitor disrupts IL-6 mediated induction of paracrine effects while leaving the extracellular release of IL-6 unaffected.

**Fig. 10:**
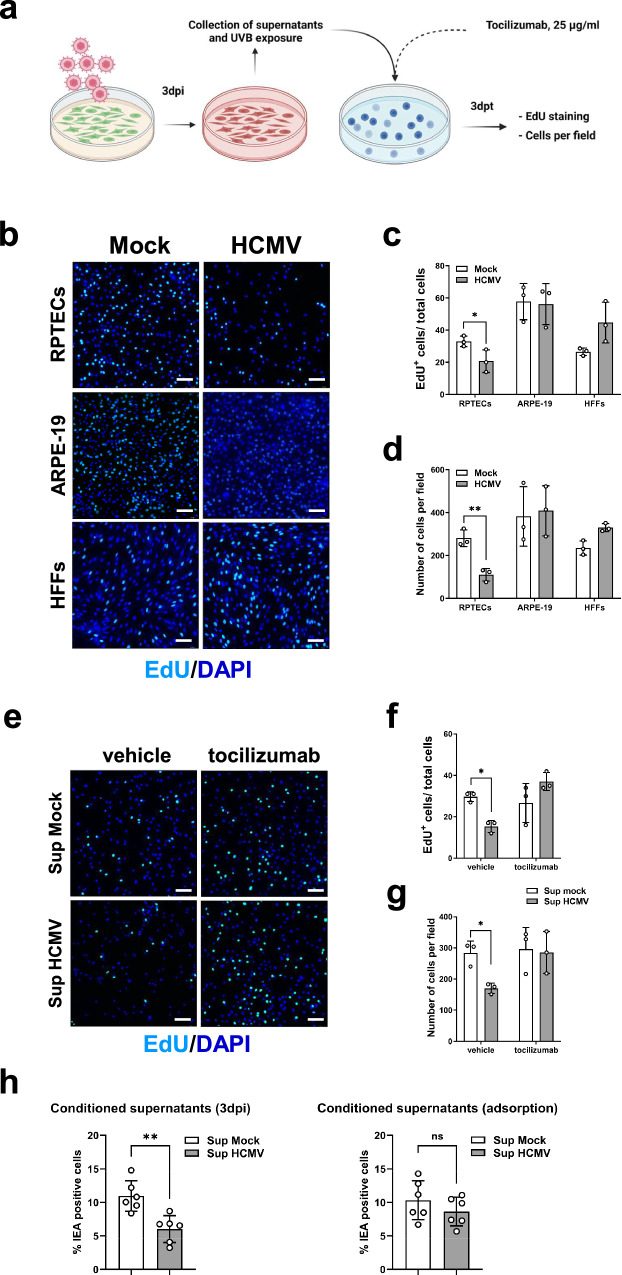
HCMV infection drives an IL-6-mediated senescence/inflammatory loop in RPTECs that is extended to bystander non-infected cells. **a** Schematic representation of the workflow followed to assess the occurrence of paracrine senescence in RPTECs, ARPE-19, and HFFs, as described in the Materials and Methods section. Created with BioRender.com. **b** Representative images of EdU incorporation staining in RPTECs, ARPE-19, and HFFs. Cells were treated for 2 days with UVB-irradiated conditioned media harvested from to the corresponding mock- or HCMV-infected cells at 3 dpi. After treatment, cells were incubated with EdU for an additional 24 h (3 days of total treatment) and stained as described in the Material and Methods section. DAPI was used to counterstain the nuclei. Scale bars, 200 μm. **c, d** Histograms representing the percentage of EdU-positive cells (**c**) or the total number of cells per field (**d**) upon treatment with UVB-exposed conditioned media from mock- or HCMV-infected cells. Three random fields of view per cell line from 3 different experimental replicates (at ×63 magnification) were captured. Data are expressed as mean values ± SD of three independent experiments (*P < 0.05, **P < 0.01; multiple unpaired t-test). **e** The conditioned medium obtained from infected RPTECs as described in **a** and **b** was used to treat fresh RPTECs in the presence of TCZ (25 μg/mL) for 48 h and then incubated with EdU for an additional 24 h, resulting in a total treatment period of 3 days. Vehicle (DMSO)-treated cells were used as control. Scale bars, 200 μm. **f, g** Histograms representing the percentage of EdU-positive cells (**f**) or the total number of cells per field (**g**) following exposure to vehicle- or TCZ-treated UVB-exposed conditioned media from mock- or HCMV-infected RPTECs. Three random fields of view per cell line from 3 different experimental replicates (at ×63 magnification) were captured and analyzed. Data are expressed as mean values ± SD of three independent experiments (*P < 0.05; multiple unpaired t-test). h RPTECs were infected with HCMV (MOI 1) and cultured for 3 days with the conditioned media generated as described in **a** (left panel) or adding fresh medium after the first 24 hpi (right panel). The results representing the percentage of IEA-positive infected RPTECs, normalized to the total number of DAPI-positive cells, were plotted. Image analysis was conducted using LAS X software (Leica Microsystems) and values are expressed as mean ± SD (error bars) relative to six different experiments (**P < 0.01; multiple unpaired t-test).

Next, we asked whether the conditioned medium from HCMV-infected cells would be able to propagate a senescence response to freshly seeded cultures. To this end, supernatants collected from RPTECs, HFFs, and ARPE-19 cells at 3 dpi were subjected to UVB irradiation to eliminate the virus-depleted and avoid re-infection, as confirmed by standard plaque assay (Supplementary Fig. 6), and then added to newly seeded cells for three days (Fig. 10a). As depicted in Fig. 10b,c, the total amount of EdU incorporation and the overall cell count per field were significantly reduced in RPTECs (∼21% *vs*. 33%, *P* < 0.05 and 110 *vs*. 280, *P* < 0.01, respectively), while no significant changes were observed in HFFs (Fig. 10c, d, *P* > 0.05 in both cases) and ARPE-19 cells (*P* > 0.05 in both Fig. 10c, d). Finally, to confirm that the observed paracrine senescence was IL-6 dependent, we added TCZ to the UVB-inactivated conditioned medium from infected RPTECs and assessed its ability to induce paracrine senescence in fresh RPTECs. As shown in Fig. 10e,f, we failed to observe any reduction in EdU incorporation when TCZ was added to the conditioned media from mock-infected (∼27%) *vs*. HCMV-infected cells (∼34%; *P* > 0.05). Likewise, total cell count per field remained unchanged in the same experimental setup (Fig. 10g, *P* > 0.05).

To determine whether the senescent phenotype induced by the virus in RPTECs is part of the pro-viral response or a host defense mechanism, we treated infected RPTECs with conditioned medium harvested at 3 dpi from either infected or mock-infected RPTECs, followed by IEA staining. Figure 10h shows a significant reduction in IEA positivity in cells exposed to the conditioned medium from infected cells when compared to those treated with supernatants from mock-infected cells. Notably, removing the conditioned media from infected cells after 24 h of exposure restored the expected number of IEA-positive cells, indicating that sustained presence of SASP is required to inhibit viral infection. These findings suggest that HCMV-induced senescence in RPTECs is more likely as a host anti-viral mechanism than a pro-viral strategy.

Overall, these findings further elucidate the differential response to HCMV infection in various cell types. While HCMV infection triggers senescence in both renal cells and fibroblasts, it specifically evokes a pronounced SASP signature and paracrine antiviral effects in renal epithelial cells. This distinct response appears to be mediated by IL-6, underscoring the unique cellular mechanisms at play in renal epithelial cells in the context of HCMV infection.

### Presence of senescence-associated markers in HCMV-infected human tissues

As it is quite challenging to precisely identify HCMV-infected cells in samples from HCMV viremic individuals, such as kidney biopsies from KTRs, we decided to assess the cellular localization of HCMV in human tissues using formalin-fixed paraffin-embedded (FFPE) tissue samples from a preterm newborn delivered by a woman who had experienced acute HCMV infection, which had led to preterm delivery and subsequent rapid death of the newborn^49^. Tissue sections from various organs were stained with an antibody cocktail targeting different HCMV antigens commonly used for diagnostic purposes. As shown in Supplementary Fig. 5a, both kidney tubular cells—mostly from proximal tubules (black arrowheads)—and squamous epithelial cells in the parietal layer of the Bowman’s capsule (black arrows) were strongly stained with anti-HCMV antibodies, whereas glomerular endothelial cells were not. Notably, many HCMV-infected cytopathic cells, especially those in the tubules (Supplementary Fig. 5b left panel, black arrowheads), displayed massive cytoplasmic accumulations of hyper-pigmented lipofuscin (LF) granules, which were well evident upon H&E staining (Supplementary Fig. 5b right panel, black arrows). LF granules are lysosome-derived insoluble aggregates of oxidized and modified lipids or proteins, typically present in damaged-senescent cells^50^. Being LF a well-regarded hallmark of senescent cells in FFPE tissues^51^, its presence suggests that HCMV can drive epithelial cells toward senescence also in its natural setting.

When examining other tissues, including the liver, pancreas, lung, brain, and testis, we found that the majority of HCMV-infected cells were of epithelial origin—*e.g*., pneumocytes, hepatocytes, and pancreatic ductal cells and those populating Langerhans’ islets (Supplementary Fig. 7). Fittingly, we detected the presence of LF granules in HCMV^+^ cells from the pancreas and in some HCMV^+^ hepatocytes, while they were barely detectable in other organs, including the lung, where the pneumocytes were heavily infected. Finally, we failed to detect the presence of HCMV^+^ fibroblasts in any of the organs analyzed.

Altogether, these data seem to indicate that epithelial cells, rather than fibroblasts, are involved in HCMV-mediated pathogenesis in vivo, and that induction of senescence may lead to virus-related disease, at least in certain organs, such as the kidney.

## Discussion

In this study, we present compelling in vitro evidence showing that RPTECs fully support HCMV replication, which in turn triggers a cellular senescence program characterized by growth arrest and induction of pro-inflammatory cytokines. Remarkably, we show that HCMV-infected RPTECs—but not similarly infected HFFs or ARPE-19 cells—display a harmful secretory phenotype that induces IL-6-dependent paracrine senescence in neighboring uninfected epithelial cells, suggesting that HCMV-evoked senescence may specifically contribute to antiviral mechanisms and the pathogenesis of kidney disease.

In the first part of our study, we show that RPTECs can support a complete lytic replication cycle of HCMV, display the canonical virus-induced CPE, and produce virus titers in their culture supernatants, albeit to a lesser extent compared to virus-infected ARPE-19 cells, an epithelial cell line known for its permissiveness to HCMV replication^35^. However, a comprehensive analysis of the transcriptome reveals profound differences among the three cell lines. While both HCMV-infected RPTECs and HFFs display somewhat similar signatures of response to the virus and type I interferon, these signatures are absent in ARPE-19 cells. Of note, only infected RPTECs are characterized by a robust inflammatory gene signature very much resembling SASP^42^, with paradigmatic inflammatory cytokines (*i.e*., IL-6, IL-1β, CXCL8/IL-8, CCL5/RANTES and CXCL10/IP-10) significantly upregulated. The functional relevance of this inflammatory signature is further supported by our Bio-Plex analysis, which reveals a massive release of inflammatory cytokines in the supernatants of infected RPTECs. In particular, TNF-α, IL-6, IL-8, and IL-9 are significantly upregulated at 1 dpi and remain elevated for up to 5 dpi, whereas GM-CSF, MIP1α, IP-10, PDGF-BB, and RANTES progressively increase overtime peaking at 5 dpi, with the sole exception of TNF-α, plateauing at 4 dpi. Finally, consistent with a picture of renal epithelial cell dysfunction, HCMV-infected RPTECs show increased expression and secretion of CXCL10/IP-10 and NGAL, known biomarkers of acute tubular injury and kidney graft rejection^40,41,52^.

Leveraging on these results, we speculated that HCMV-infected RPTECs may exhibit features of VIS^25,26^. It is in fact well-established that both DNA and RNA viruses can induce classic senescence hallmarks, such as SA-β-gal activity, increased expression of p16^INK4a^, and p21^Waf1^, as well as the secretion of pro-inflammatory SASP molecules, and the generation of reactive oxygen species (ROS)^53–56^. Furthermore, previous in vitro studies on fibroblasts have shown that viral infection affects the host cell cycle and results in cytoplasm enlargement, induction of replication stress, and release of pro-inflammatory cytokines^15,30,57^. In our earlier work, we reported p53- and p16^INK4a^-mediated premature senescence in HCMV-infected primary human fibroblasts. Fittingly, the overexpression of the viral 86-kDa IE2 protein inhibited cellular DNA synthesis, upregulated SA-β-gal expression, and induced morphological changes, such as the acquisition of an enlarged, flattened, and irregular shape, all typical hallmarks of senescent cells^29,58^. Thus, these findings corroborate the notion that HCMV infection can induce senescence in RPTECs, highlighting the remarkable similarity in VIS programs observed across various cell types.

Despite the emerging role of VIS in infectious disease pathogenesis, the biological relevance of senescence in the context of HCMV infection—likely involving epithelial cells rather than fibroblasts—remains largely unexplored^29^. Thus, in the second part of the study, we sought to determine the specific cellular events occurring in RPTECs *vs*. those taking place in fibroblasts or in another epithelial cell line such as ARPE-19 following HCMV infection. Using the newly established senescence-specific gene set SenMayo, we found a significant enrichment of this senescence signature in HCMV-infected RPTECs, which was less pronounced in HFFs and basically absent in ARPE-19 cells. Moreover, both HCMV-infected RPTECs and HFFs displayed additional standard senescence markers, such as enlarged cytoplasm, increased SA-β-gal expression, and reduced proliferation, which were not found in ARPE-19 cells. Despite this common signature, further analysis revealed distinct features that characterize the response of RPTECs to HCMV infection, including downregulation of *Ki67* and *Lamin B1* gene expression, also indicative of a senescent state^45^. Likewise, a much higher amount of IL-6 and IL-8 was released by HCMV-infected RPTECs compared to similarly infected HFFs, while ARPE-19 production of both cytokines was negligible.

The RPTEC host reaction to HCMV infection was also marked by the presence of nuclear NF-κB, γH2AX, and p16^INK4a^ in the nuclei of a significant number of neighboring uninfected cells, raising the possibility that virus-mediated paracrine senescence might be specific to renal tubular cells. Consistently, our results using UVB-inactivated conditioned medium from infected cells confirmed that only RPTEC-conditioned medium, but not that from HFFs or ARPE-19 cells, significantly reduced the percentage of EdU-positive cells per field as well as the total cell count per field.

The emerging concept of senescence and paracrine senescence as an indirect amplification mechanism of virus-induced damage^47^ has been observed in other viral infections, including SARS-CoV-2^28,59^. We speculate that a similar mechanism may be at play in HCMV-infection, particularly in renal tubular cells.

While several inflammatory cytokines have been collectively involved in the induction of paracrine senescence, our results strongly support the hypothesis that IL-6 is involved in the induction and maintenance of paracrine senescence in our model of HCMV-infected RPTECs. Indeed, our transcriptome analysis revealed a specific enrichment of the IL-6/JAK-STAT3 signaling pathway in HCMV-infected RPTECs, which led us to demonstrate that exposure to the IL-6R inhibitor TCZ can significantly disrupt the paracrine inflammatory loop elicited by HCMV infection. Addition of TCZ during infection resulted in a marked reduction in the total number of cells expressing nuclear NF-κB and γH2AX compared to infected vehicle-treated cells, particularly in bystander uninfected cells. When TCZ was added to the UVB-inactivated conditioned medium from infected RPTECs, it significantly increased the percentage of EdU-positive cells, indicating a restoration in cellular proliferation. This suggests that the ability of the conditioned medium to trigger senescence in fresh cells is primarily mediated by factors, such as IL-6, which are susceptible to neutralization by TCZ. Correspondingly, exposure of RPTECs to the conditioned medium from infected RPTECs significantly impaired viral replication, as judged by the number of IEA-positive cells. Consequently, we propose a model wherein IL-6 plays an essential role in initiating and promoting paracrine senescence in response to HCMV infection. This mechanism is integral to limiting the spread of HCMV to adjacent, uninfected cells.

Further supporting our in vitro results, immunohistochemistry (IHC) of FFPE tissue sections derived from a full-blown HCMV infection in a preterm child revealed a large number of epithelial cells from different organs (*i.e*., liver, kidney, and pancreas) expressing HCMV proteins and exhibiting significant accumulation of LF aggregates. These granules consist of metals, misfolded proteins, and lipid-based autofluorescent pigments that localize in senescent lysosomes^60^. Importantly, LF aggregate-expressing kidney proximal tubular cells were those more frequently affected by virus-related morphological changes, reinforcing the significance of HCMV-induced senescence in the kidney compartment.

It is important to acknowledge that this observation is limited to only one case, and we did not perform co-staining for viral proteins and senescence markers. Nevertheless, the presence of LF granules in cells displaying virus-induced cytopathic effects suggests the potential existence of HCMV-induced senescence also in the in vivo setting.

An important question raised by our results is whether HCMV gains an evolutionary advantage by driving cells into senescence. Although our findings do not clarify whether VIS favors viral persistent infection in growth-arrested but metabolically active epithelial cells, or if it is merely an incidental event that does not impact viral fitness, they provide the first evidence of a direct pathogenic mechanisms of HCMV infection in the kidney involving the induction of cellular senescence in a paracrine manner.

Further research is clearly needed to fully understand the relevance of senescence in HCMV-mediated disease in vivo.

## Materials and Methods

### Cells, viruses, and treatments

Human foreskin fibroblasts (HFFs, ATCC SCRC-1041) and ARPE-19 cells (ATCC CRL-2302) were cultured in high glucose Dulbecco’s modified Eagle’s medium (DMEM, Sigma-Aldrich, Milan, Italy) supplemented with 10% fetal bovine serum (FBS, Sigma-Aldrich) and 1% of a penicillin-streptomycin-gentamycin solution (Sigma-Aldrich). hTERT-immortalized human renal proximal tubular epithelial cells (RPTEC/TERT1, Evercyte GmbH, Wien, Austria) were grown in ProxUp Supplement media (MHT-003-2, Evercyte GmbH). The HCMV TR laboratory strain was propagated through two passages in ARPE-19 cells and one passage in HFFs before being titrated on HFFs using a standard plaque assay^61^. UVB-inactivated HCMV TR was prepared using a double pulse of UVB light (1.2 J/cm2). For each experiment, 5 × 10^4^ (RPTECs and ARPE-19) or 7 × 10^4^ (HFFs) cells were seeded in 6-well plates and, after 24 h, infected with HCMV TR strain at an MOI of 1, 3 and 0.5, respectively. The infection was stopped after 24 h by changing the media. Cells were observed from 1 dpi to 8 dpi, considering the time of infection as day zero. For experiments with conditioned medium, the infections were stopped at 3 dpi. The supernatants were collected, centrifuged at 10,000 g for 5 min, treated using double pulse of UVB light (1.2 J/cm2), and used to treat freshly seeded cells for an additional 3 days. Where described, the IL-6R inhibitor TCZ (HY-P9917; MedChemExpress, Monmouth Junction, NJ, USA) was added to the cells at each media change, starting from the day of infection or treatment with conditioned medium, at a concentration of 25 μg/ml.

### Immunofluorescence microscopy

RPTECs (1.5 × 10^4^), ARPE-19 (2.5 × 10^4^), and HFFs (3.5 × 10^4^) were seeded in triplicate on coverslips in 24-well plates and infected with HCMV TR at an MOI of 1, 3 and 0.5, respectively. At the indicated time points, cells were fixed with 4% paraformaldehyde and then incubated for 1 h using appropriate dilutions of primary antibody in a dark humidified chamber at room temperature (RT), followed by secondary labeled antibody for 1 h. DAPI (4′,6′-diamodino-2phenylindole) (D1306, Thermo Fischer Scientific, Waltham, MA, USA) was used to counter-stain the nuclei. The following primary and secondary antibodies were used: monoclonal anti-HCMV IEA (P1215, Virusys Corporation, Taneytown, MD, USA) or in house-produced rabbit polyclonal anti-IEA^62^, monoclonal anti-HCMV pp28 (P1207, Virusys Corporation), monoclonal anti-HCMV UL44 (P1202, Virusys Corporation), monoclonal NF-κB (L8F6 6956, Cell Signaling Technology, Danvers, MA, USA), rabbit monoclonal p16^INK4a^ (E6N8P) (18769 S, Cell Signaling Technology), γH2AX (JBW301, Sigma-Aldrich), goat anti-mouse IgG-Alexa Fluor 488 (A11001, Thermo Fisher Scientific), and goat IgG anti-Rabbit IGA-Alexa Fluor (A-11036, Thermo Fisher Scientific). The coverslips were mounted with SlowFade Gold antifade reagent mounting media (Thermo Fischer Scientific), and cells visualized using a Leica Thunder Imager 3D System (Leica Microsystems, Wetzlar, Germany). The percentage of infected cells expressing IEA, UL44, and pp28 and the percentage of positive cells displaying NF-κB, p16^INK4a^ and γH2AX for each cell line were normalized to the total number of DAPI-positive cells. Image analysis was carried out using the LAS X software (Leica Microsystems), and values were expressed as mean ± SD (error bars).

### EdU (5-ethynyl-2′-deoxyuridine) staining

For the proliferation assay, cells were seeded and infected following the same protocol as described for immunofluorescence microscopy. Additionally, cells were cultured with UVB-inactivated conditioned medium, as previously mentioned, for 3 days. At day 2 post-infection or post-treatment, cells were treated with 50 µM of EdU for 20 h. Cells were then fixed, stained with the ClickTech EdU Cell Proliferation Kit 488 (baseclick GmbH, Neuried, Germany) and counterstained with DAPI according to the manufacturer’s instructions. Images were acquired using a THUNDER Imager Live Cell & 3D system (Leica Microsystems) using the Navigation tool (63x magnification). For each sample, 16 fields were quantified in triplicates. The number of cells was counted through LAS X software (Leica Microsystems). The images were cropped using Microsoft PowerPoint (Microsoft 365, Version 2206, 2019).

### Plaque assay

To determine the infectivity of the supernatants, a standard plaque assay was carried out on HFFs^61^. Briefly, 2.5 × 10^4^ HFFs were seeded in 96-well plates in DMEM supplemented with 10% FBS. Supernatants from HCMV-infected RPTECs, ARPE-19 or HFFs—diluted from 10^−1^ to10^−7^—were then added to HFFs in duplicate, centrifuged at 3000 rpm for 30 min, and incubated for 2 h at 37 °C. Supernatants were then discarded, and cells were layered with 0.8% methylcellulose. After 8 days, the methylcellulose layer was removed, and the cells were stained with 0.1% crystal violet, incubated at RT for 30 min on a shaker, washed with water, and dried. The plaques were manually counted under a microscope.

### RNA extraction and library construction

For transcriptome analysis, mRNA was extracted using the miRNeasy Tissue/Cells Advanced Kit (Qiagen, Hilden, Germany). Samples quality was confirmed with Agilent 4200 TapeStation system and HS D1000 RNA kit (Agilent, Santa Clara, CA, USA), with all samples showing an RNA Integrity Number (RIN) > 8, indicating good quality of the extracted material. Library preparation was performed with an Illumina TruSeq Stranded mRNA kit (Illumina, Inc., San Diego, CA, USA) using 500 ng of total RNA per sample. mRNA sequencing was then performed on a NextSeq 550 platform (Illumina, Inc.) through NextSeq 500/550 High Output Kit v2.5 (150 Cycles - 2 × 75 read length, paired-end) (Illumina, Inc.).

### Transcriptomic analysis

Total RNA from infected and parental RPTECs was isolated and subjected to high-throughput sequencing to analyze poly-A^+^ RNAs. Two biological replicates were analyzed for each sample. RNA sequencing was performed with an Illumina NextSeq550 sequencer (Illumina, Inc.). In parallel, paired-end raw reads of parental and infected HFF and ARPE-19 cells were downloaded from GSE120891. These reads, together with those from parental and infected RPTECs, were analyzed using the following pipeline: The STAR program^63^ was used to align reads to the reference human hg38 genome. Ensembl v100 annotations were set as the reference for the RSEM computational pipeline^64^, which was employed to quantify gene expression levels. DEGs were calculated using DESeq2^65^ with parameters of log2 fold change (log2FC) > 2 and adjusted p-value (p.adj) < 0.05 to determine the statistical significance of DEG. All statistical and graphical computations were performed in the R environment (https://www.r-project.org/). Unsupervised hierarchical clustering of genes was visualized as a heatmap using the “pheatmap” package in R, with expression values presented as log2. GSEA was employed to investigate the cellular response to HCMV infection^66^. Gene Set Enriched Analysis was performed using the desktop application and significant GSEA terms were filtered using the following cut-offs: |NES | > 1 and FDR < 0.05. Functional analyses of DEGs were performed using Metascape^67^ with default parameters.

### Quantitative real-time PCR

Quantitative real-time PCR (qRT-PCR) was performed with a CFX96 Real-Time PCR Detection System (Bio-Rad Laboratories, Hercules, CA, USA), as previously described^68^. Total RNA was extracted using TRI Reagent (Sigma-Aldrich), and 1 μg was retrotranscribed using an iScript cDNA Synthesis Kit (Bio-Rad). Reverse-transcribed cDNAs were amplified in duplicate using SsoAdvanced Universal SYBR Green Supermix (Bio-Rad Laboratories). The 18 S gene was used as housekeeping gene for normalization of cDNA levels. The relative normalized expression after stimulation was calculated as fold change over control using the formula = 2 -Δ(ΔCT) where ΔCT = CTtarget—CT18S and Δ(ΔCT) = ΔCTstimulated - ΔCTcontrol. The primer sequences used in qRT-PCR experiments were as follows: LaminB1FW 5′-AAG CAT GAA ACG CGC TTGG-3′, LaminB1REV: 5′-AGT TTG GCA TGG TAA GTC TGC-3′; Ki67FW 5′-TCC TTT GGT GGG CAC CTA AGA CCT G-3′, Ki67REV 5′-TGA TGG TTG AGG TCG TTC CTT GAT G-3′; 18SFW 5′-TCC CCA GCC CTT TTG TTG A, 18SREV 5′-TTA GAA CCA AAT GTG GCC GTG-3′.

### Multiplex immunoassay of cytokines/chemokines

Supernatants collected from infected or mock-infected RPTECs were screened for the presence of 27 human cytokines and chemokines using the Bio-Plex ProTM Human Cytokine Grp I Panel 27-Plex kit (Bio-Rad Laboratories). The analysis was performed with Bio-Plex 200 System (Bio-Rad Laboratories) according to the manufacturer’s instructions.

### LCN2/NGAL, IL-6 and IL-8 measurement by ELISA

Cytokines secreted in culture supernatants after treatments were analyzed using the human LCN2/NGAL ELISA kit (Hycult Biotech, Uden, NL), human IL-6 DuoSet ELISA and human IL-8 DuoSet ELISA (all from R&D Systems) according to the manufacturer’s instructions. Absorbance was measured using a Spark multimode microplate reader (Tecan, Männedorf, Switzerland).

### SA-β-gal staining

For SA-β-gal detection, RPTECs (1.5 × 10^4^), ARPE-19 (2.5 × 10^4^), and HFFs (3.5 × 10^4^) were seeded in 24-well plates. At 3 dpi, cells were washed in PBS and then fixed for 4 min at RT with 0.3 ml of fixative solution (2% formaldehyde, 0.2% glutaraldehyde diluted in PBS). Fixed cells were washed twice with PBS, incubated overnight at 37 °C in 0.5 ml of staining solution (see below), without providing CO^2^, and then stained with DAPI for 30 min before acquisition. Raw images (2 × 2 montage) were acquired using a 4X objective with a Cytation 5 Cell Imaging Multi-Mode Reader (Agilent BioTek, Santa Clara, CA, USA), in both the fluorescent and bright-field channels to visualize nuclei and SA-β-gal positive cells, respectively, and then processed and stitched using the default setting^69^ using the proprietary software. The staining solution was prepared in deionized water with 1 mg/mL of 5-bromo-4-chloro-3-indolyl-beta-d-galactopyranoside (X-gal, Thermo Fischer Scientific), 1× citric acid/sodium phosphate buffer, 5 mM potassium ferricyanide, 5 mM potassium ferrocyanide, 150 mM NaCl and 2 mM MgCl_2_.

### Western blot analysis

Whole-cell extracts were prepared using RIPA lysis and extraction buffer (Pierce) with halt protease and phosphatase inhibitor (Thermo Fisher Scientific) on ice, and total protein concentration was quantified by Bradford Reagent (Sigma-Aldrich) measuring absorbance at 595 nm. Twenty μg of cell extracts, were separated by electrophoresis on 7.5% or 12% SDS-polyacrylamide gels (Bio-Rad), transferred to nitrocellulose membranes, blocked with 10% non-fat milk in tris-buffered saline-tween (TBST), and probed with specific primary antibodies O/N at 4 °C. The following primary and secondary antibodies were used: monoclonal anti-HCMV IEA (P1215, Virusys Corporation) or in house-produced rabbit polyclonal anti-IEA^59^, monoclonal anti-HCMV pp28 (P1207, Virusys Corporation), monoclonal anti-HCMV UL44 (P1202, Virusys Corporation). After being washed with TBST, membranes were incubated with specific HRP-conjugated secondary antibodies, and binding was detected by ECL (Thermo Fisher Scientific, Super Signal West Pico). Expression of β-actin was used as protein loading control.

### Histological examination of human tissues

All samples were subjected to H&E staining to detect signs of cytopathic damage (*i.e*., inclusion bodies). IHC staining for HCMV was performed on all formalin-fixed paraffin-embedded (FFPE) samples, sectioned at 4 μm on polarized slides. HCMV IHC was performed using a Benchmark ULTRA staining module (Ventana Medical Systems, Roche Diagnostics, MB, Italy) equipped with a fully automated protocol. Briefly, slides—previously baked overnight at 37 °C—were deparaffinized and rehydrated. Antigen retrieval was achieved using UltraCC1 (Ventana Medical Systems) for 36 min. The prediluted HCMV antibody (anti-HCMV blend cocktail 8B1.2, 1G5.2 & 2D4.2 mouse monoclonal primary antibody; catalogue No. 760-4703; Ventana Medical Systems) was incubated for 32 min at RT. Signal amplification was performed with the Amplification kit (Ventana Medical Systems), and detection was carried out with UltraView Universal DAB Detection kit (Ventana Medical Systems). To enhance visualization, counterstaining was performed with hematoxylin for 8 min, followed by 4-min incubation with Bluing Reagent (Ventana Medical Systems). Upon completion of the automated staining, the slides were subjected to dehydration and coverslipping through an HE600 platform (Ventana Medical Systems; Roche Diagnostics).

### Viral entry assay

RPTECs, HFFs and ARPE-19 were sub-confluently plated in a 6-well plate. HCMV at MOI 1, 0.5 and 3, respectively, was adsorbed for 3 h at 4 °C on pre-chilled cells to allow viral attachment. Cells were then washed with cold medium three times to remove unbound virus and incubated for 1 h at 37 °C for virus entry. The unpenetrated virus was inactivated with acidic glycine for 2 min at room temperature. Cells were then washed with warm medium three times to return the pH to neutral. Total DNA was extracted using the PureLink Genomic DNA Mini Kit (Life Technologies) according to the manufacturer’s instructions. Finally, cell-penetrated HCMV genome copy numbers were quantified using the QX200 Droplet Digital PCR System (Bio-Rad) on 10 ng of DNA sample and expressed as IE1 and UL54 copy numbers. The 18 S gene was used as a housekeeping gene for normalization. The primer sequences used in ddPCR experiments were as follows: UL54FW 5′-TAC GAG GTA GCC GAA GAT CC-3′, UL54REV 5′-GGC GAC AGC ACG TTA GTT AC-3′; IE1FW 5′-AAG CGG CCT CTG ATA ACC AAG, IE1REV 5′-GAG CAG ACT CTC AGA GGA TCG-3′; 18SFW 5′-TCC CCA GCC CTT TTG TTG A, 18SREV 5′-TTA GAA CCA AAT GTG GCC GTG-3′.

### PI permeabilization assay

The cell death ratio was calculated as previously described^70^. Briefly, RPTECs (1.5 × 104), ARPE-19 (2.5 × 104), and HFFs (3.5 × 104) were seeded in triplicate in 24-well plates and infected with the HCMV TR strain at an MOI of 1, 3 and 0.5, respectively. After 3 dpi, cells were washed three times with PBS, then 300 μl of pre-warmed staining solution (5 μM PI, 5% FBS, 20 mM HEPES in HBSS without phenol red but with MgCl2 and CaCl2) was added to each well and incubated for 5 min at 37 °C 5% CO2. As a positive control for maximum permeability, 0.1% Triton X-100 was used. The fluorescence intensity was measured using a Spark multimode microplate reader (Tecan, Männedorf, Switzerland), equipped with 544 nm excitation and 620-10 nm emission filters.

### Statistics and reproducibility

All experiments were repeated at least three times, with the exception of the transcriptomic analysis in which two biological replicates were analyzed for each sample. All statistical analyses were performed using GraphPad Prism version 9.20 for Windows (GraphPad Software, La Jolla California USA, www.graphpad.com). The data were presented as mean ± SD. Statistical significance was determined by unpaired t test (two-tailed) or two-way analysis of variance (ANOVA) with Dunnett’s post-test. Differences were considered statistically significant for *P* < 0.05 (*P < 0.05; **P < 0.01; ***P < 0.001; ****P < 0.0001).

### Reporting summary

Further information on research design is available in the Nature Portfolio Reporting Summary linked to this article.

### Supplementary information

Supplementary information

Description of Additional Supplementary Files

Supplementary Data 1

Reporting summary

## Data Availability

The source data behind the graphs in the manuscript were shown in Supplementary Data 1, and the list of DEGs in RPTECs are available in Supplementary Table 1. Uncropped gels for Fig. 1b can be found under Supplementary Information as Supplementary Fig. 8. All other relevant data are available from the corresponding authors upon reasonable request. Raw reads and processed sequencing data have been deposited in the NCBI Gene Expression Omnibus and are publicly available under the accession number GSE185442.
