# Supplementary material for: Human cytomegalovirus infection triggers a paracrine senescence loop in renal epithelial cells
Source: Commun Biol. 2024 Mar 8;7:292. doi: 10.1038/s42003-024-05957-5 (PMC10924099; doi:10.1038/s42003-024-05957-5)
Supplement: Supplementary file 1 — Supplementary information [file 42003_2024_5957_MOESM1_ESM.pdf]

Human cytomegalovirus infection triggers a paracrine senescence loop in renal epithelial cells

Stefano Raviola,[a,b] Gloria Griffante,[b] Andrea Iannucci,[a,b,#] Shikha Chandel,[b] Irene Lo Cigno,[b] Davide Lacarbonara,[a,b] Valeria Caneparo,[a] Selina Pasquero,[g] Francesco Favero,[c,d] Davide Corà,[c,d] Elena Trisolini,[e] Renzo Boldorini,[e] Vincenzo Cantaluppi,[f] Santo Landolfo,[g] Marisa Gariglio,[a,b,¶] Marco De Andrea,[a,g,¶,*]

[a] Intrinsic Immunity Unit and [c] Bioinformatics Unit, CAAD - Center for Translational Research on Autoimmune and Allergic Disease, University of Eastern Piedmont, Novara, Italy

[b] Molecular Virology Unit, [d] Bioinformatics Unit and [f] Nephrology and Kidney Transplantation Unit, Department of Translational Medicine, University of Eastern Piedmont, Novara, Italy

[e] Pathology Unit, Department of Health Sciences, University of Eastern Piedmont, Novara, Italy

[g] Viral Pathogenesis Unit, Department of Public Health and Pediatric Sciences, University of Turin, Medical School, Turin, Italy.

* Corresponding author:

Email: marco.deandrea@unito.it (MDA)

This file includes:

Supplementary Figure 1 – Figure 8

Supplementary Table 1

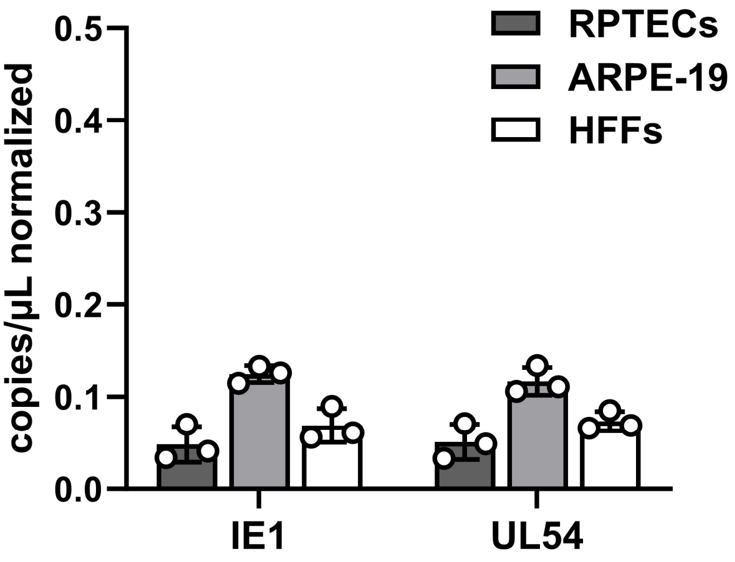

Supplementary Figure 1. HCMV viral genome quantification in RPTECs, ARPE-19 and HFFs. The viral entry assay was performed as described in the Material and Methods section. Histograms show the levels of IE1 and UL54 copies, quantified by ddPCR analysis and normalized to 18S gene copies. Data are presented as mean values ± SD from biological triplicates.

a

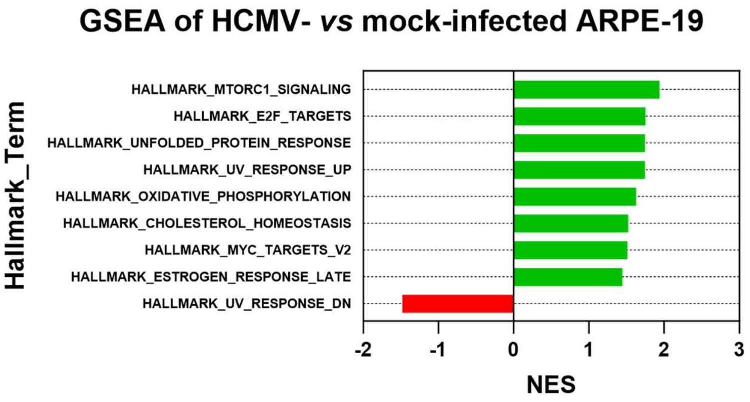

b

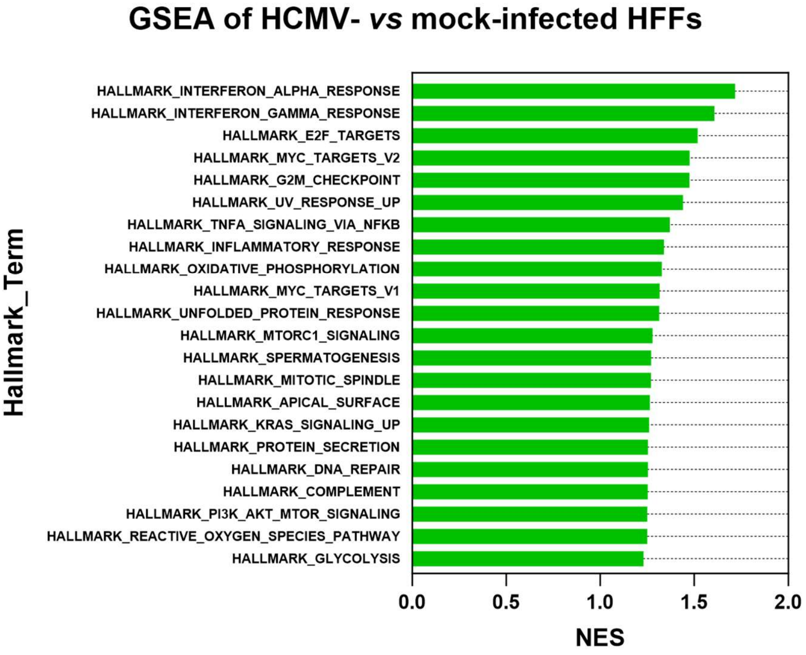

Supplementary Figure 2. Gene set enrichment analysis (GSEA) of HCMV-infected ARPE-19 (a) and HFFs (b) compared to mock-infected controls. Positive normalized enrichment score (NES) indicates enrichment (green bars), whereas negative NES indicate downregulation in virus-infected cells (red bars). NES with an FDR q-value < 0.05 are considered statistically significant and are shown.

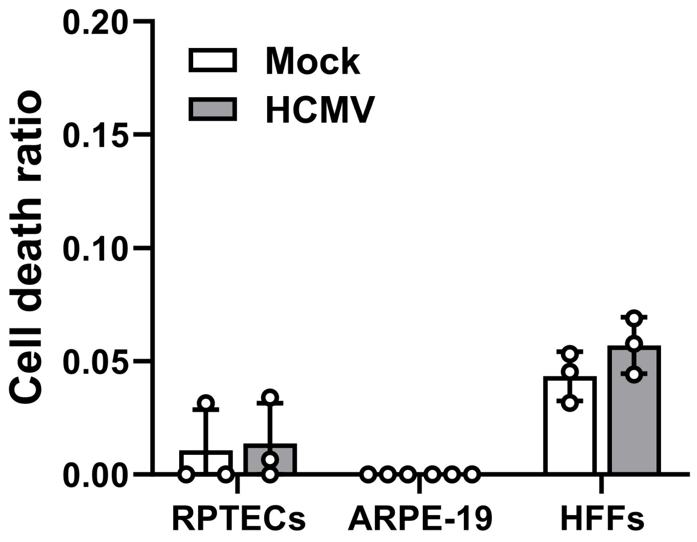

Supplementary Figure 3. HCMV infection does not increase cell death in RPTECs, ARPE-19 and HFFs. Cell viability was assessed by propidium iodide (PI) permeabilization assay as described in the Material and Methods section. The histograms show the cell death ratio in mock- or HCMV-infected RPTECs, ARPE-19 and HFFs at 3 dpi (MOI 1, 3 and 0.5, respectively). Data are expressed as mean values ± SD from three independent experiments.

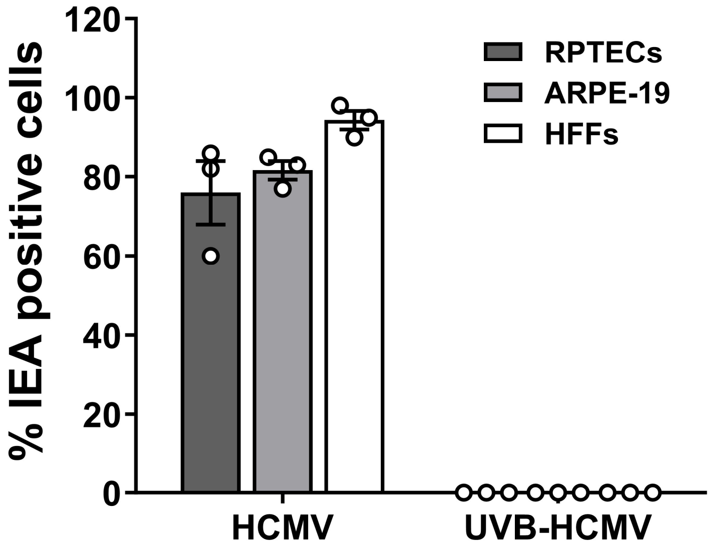

Supplementary Figure 4. Viral immediate early antigen (IEA) immunofluorescence staining demonstrating HCMV inactivation upon UVB treatment. Histograms show the percentage of cells displaying expression of IEA (at 3 dpi) for all the cell lines tested following the infection with wild type (HCMV) or UVB-inactivated (UVB-HCMV) TR strain. Data are expressed as mean values ± SD from three independent experiments.

a

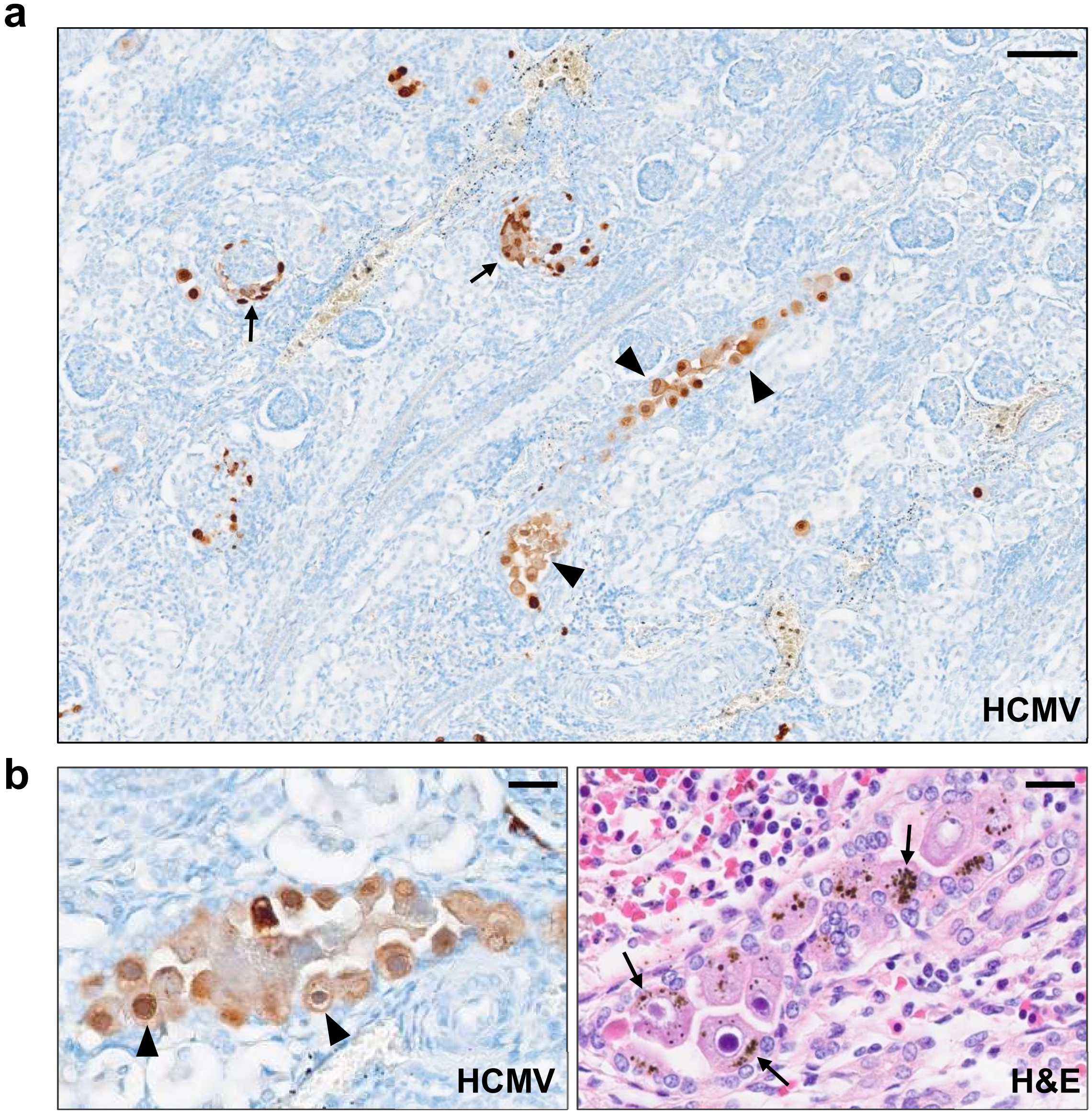

Supplementary Figure 5. Senescence-associated markers in HCMV-infected human fetal kidney. a Immunohistochemical staining was performed on formalin fixed paraffin embedded (FFPE) kidney sections from a preterm newborn delivered by a woman who experienced acute HCMV infection leading to preterm delivery and subsequent rapid death of the newborn. The sections were probed with an antibody cocktail against different viral antigens to detect HCMV reactivity. The staining primarily revealed anti-HCMV reactivity in kidney tubular cells, particularly in proximal tubules (black arrowheads) as well as in squamous epithelial cells of the parietal layer of the

Bowman's capsule (black arrows). Scale bar: 100 μm. **b** Detailed view of a proximal tubule is shown, depicting the typical cytopathic effects (CPE) associated with HCMV infection. The left panel shows immunostaining with the same anti-HCMV antibody described in A, highlighting the presence of CPE in the form of distinct changes in the tubular cells (black arrowheads). The right panel depicts the H&E staining, revealing a massive cytoplasmic accumulation of hyper-pigmented lipofuscin (LF) granules, which are insoluble aggregates of various origin derived from lysosomes typically present in damaged-senescent cells (black arrows). Scale bars: 20 μm.

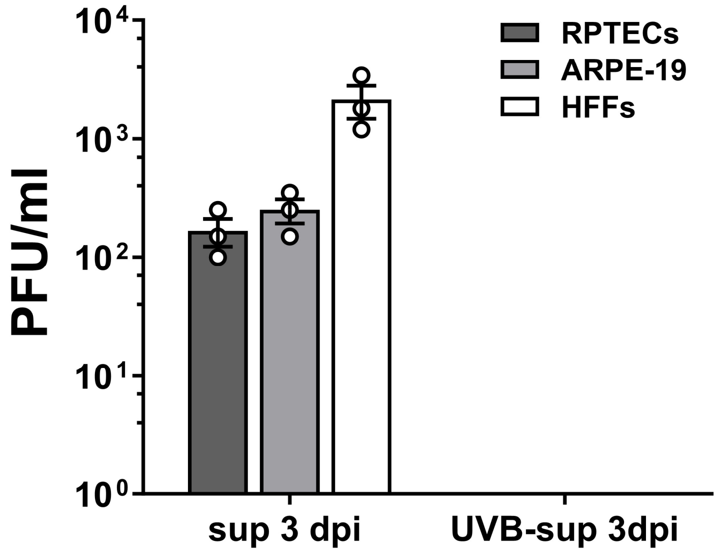

Supplementary Figure 6. Standard plaque assay demonstrating HCMV inactivation upon UVB treatment in the supernatants from infected cells. The supernatants collected from HCMV-infected RPTECs, ARPE-19, and HFFs at 3 dpi were subjected to UVB irradiation (UVB-sup 3dpi) or left untreated (sup 3 dpi). The histograms show the viral titers measured by standard plaque assay on HFFs treated with these supernatants. The plaques were microscopically counted and expressed as PFU/mL, and values are expressed as mean ± SD (error bars) from three independent experiments.

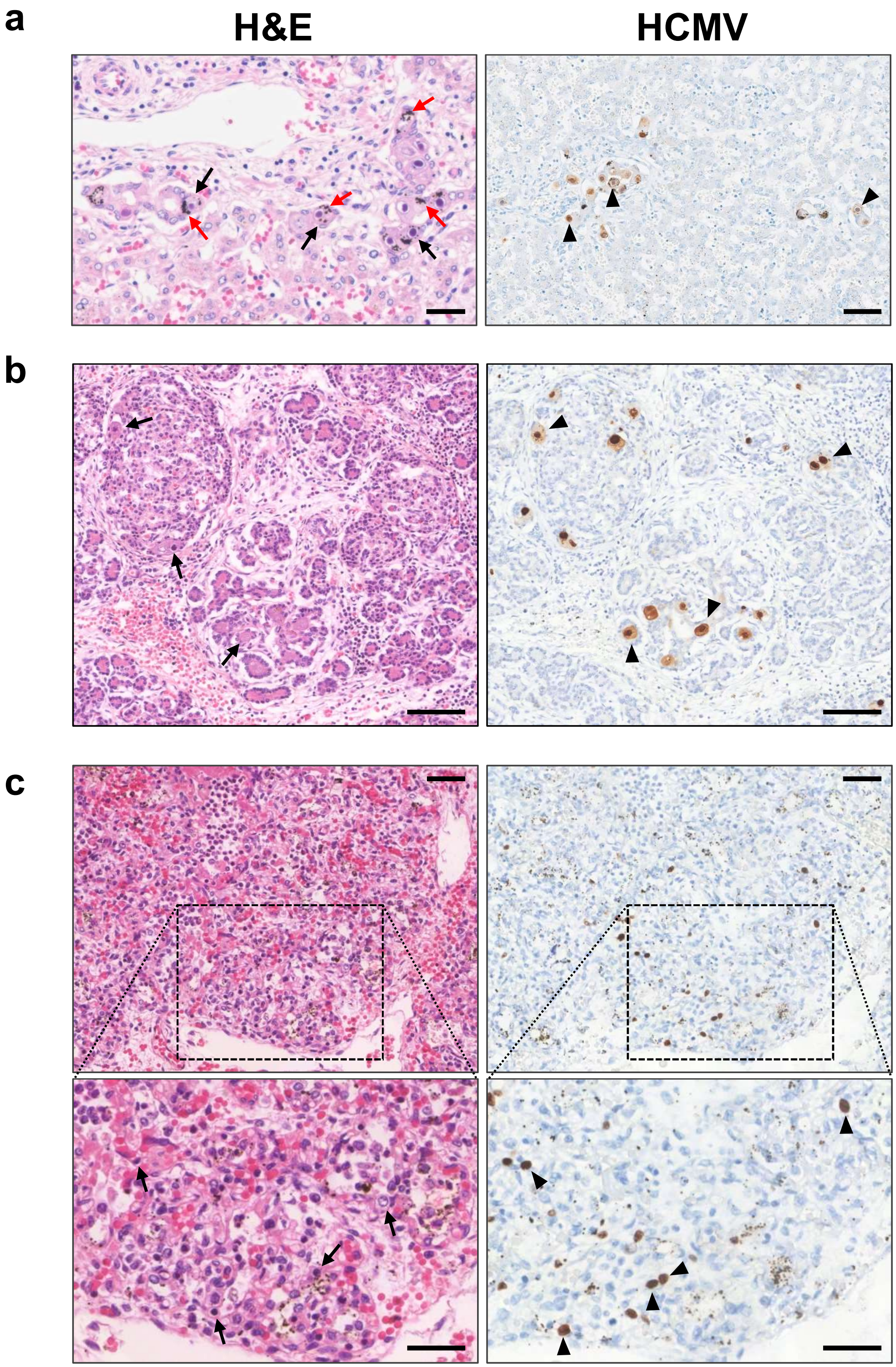
a
H&E
HCMV
b
c

Supplementary Figure 7. Senescence-associated markers in HCMV-infected human fetal tissues. H&E staining and immunohistochemical analysis were performed on fetal liver (**a**, scale bars: 100 μm), pancreas (**b**, scale bars: 100 μm), and lung (**c**, scale bars: 50 μm) sections from the same full-blown HCMV infection described in Fig 11. Anti-HCMV reactivity was detected using an antibody cocktail against various viral antigens. It can be primarily observed in cells of epithelial origin, including hepatocytes, cells lining the pancreatic ducts and those present in Langerhans' islets, and pneumocytes (arrowheads). H&E staining shows typical HCMV-associated CPE (black arrows). The deposition of LF granules, characteristic of HCMV infection, was evident in some HCMV-positive hepatocytes (**a**, red arrows)

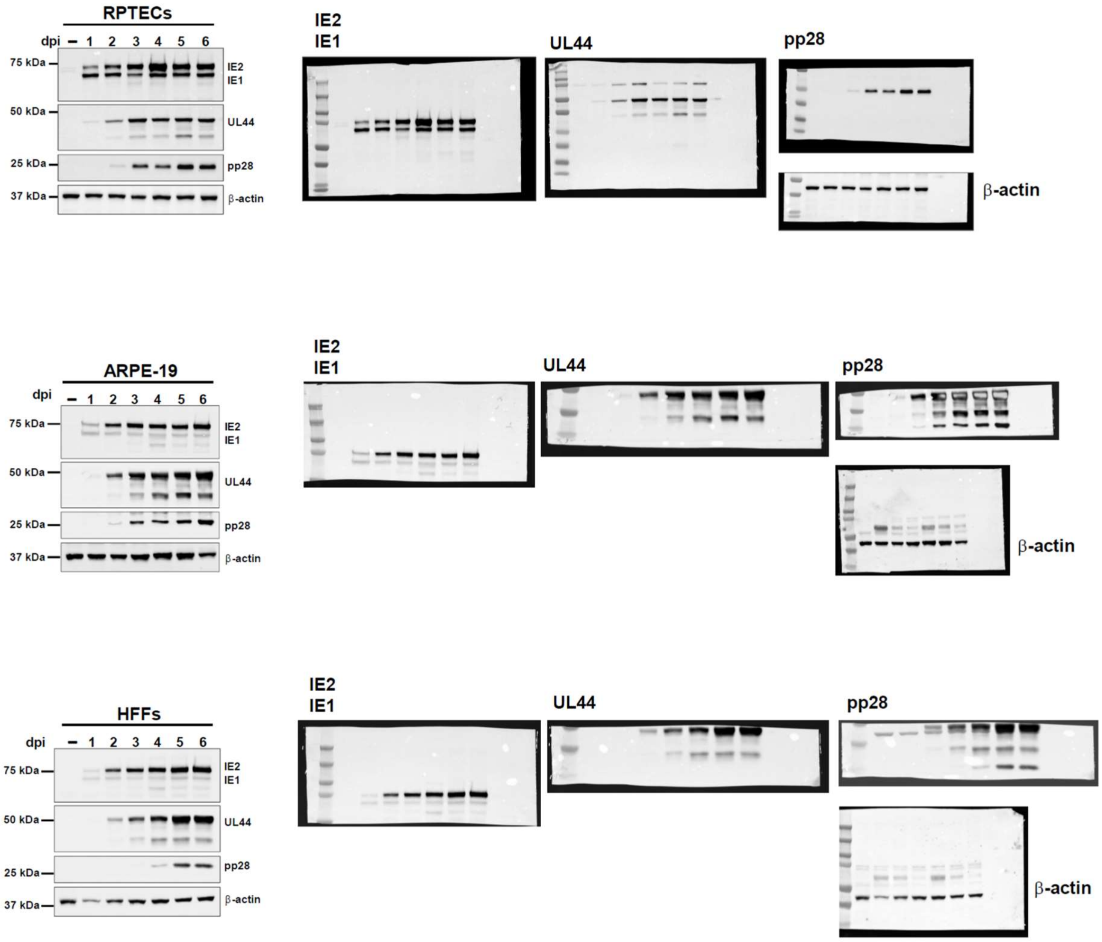

Supplementary Figure 8. Uncropped and unedited blot/gel images from Figure 1B.

Supplementary Table 1.

ENSG	Gene_name	log2FC	padj
ENSG00000008735	MAPK8IP2	7,13007	0,00024
ENSG00000010030	ETV7	2,889068	4,8E-25
ENSG00000010810	FYN	2,476979	0,0002
ENSG00000012504	NR1H4	2,264257	3,8E-05
ENSG00000028277	POU2F2	3,401059	3,7E-33
ENSG00000049323	LTBP1	2,860339	0,0031
ENSG00000055118	KCNH2	4,813714	0,03643
ENSG00000056487	PHF21B	5,57488	0,0364
ENSG00000060709	RIMBP2	5,823576	0,01951
ENSG00000064300	NGFR	3,58857	0,00103
ENSG00000065320	NTN1	2,134677	2,4E-05
ENSG00000068079	IFI35	2,06953	7E-180
ENSG00000069667	RORA	2,409908	0,02118
ENSG00000070190	DAPP1	2,882857	2,9E-33
ENSG00000070729	CNGB1	3,181486	0,02078
ENSG00000073150	PANX2	4,178613	0,0039
ENSG00000074211	PPP2R2C	4,4396	0,01068
ENSG00000076706	MCAM	2,612939	1E-08
ENSG00000078081	LAMP3	5,732045	7,7E-77
ENSG00000081041	CXCL2	3,095539	3,7E-45
ENSG00000081248	CACNA1S	6,37953	0,0003
ENSG00000087085	ACHE	4,640999	0,00337
ENSG00000088386	SLC15A1	4,122328	2,5E-07
ENSG00000089127	OAS1	4,537828	0
ENSG00000090659	CD209	2,694586	0,0216
ENSG00000093134	VNN3	6,218934	0,00597
ENSG00000095752	IL11	3,133085	6E-06
ENSG00000100167	SEPTIN3	4,514152	2E-06
ENSG00000100234	TIMP3	2,598469	0,00075
ENSG00000100321	SYNGR1	3,717615	0,02616
ENSG00000100628	ASB2	3,724664	1,5E-29
ENSG00000101096	NFATC2	6,967789	0,00045
ENSG00000101134	DOK5	7,098951	0,00026
ENSG00000101210	EEF1A2	4,310333	1,6E-21
ENSG00000101335	MYL9	3,275371	2,2E-10
ENSG00000101347	SAMHD1	2,080595	0
ENSG00000103742	IGDCC4	2,572973	7,7E-68
ENSG00000104213	PDGFRL	2,056817	0,00782
ENSG00000104951	IL4I1	2,320314	1E-78
ENSG00000105088	OLFM2	5,654041	0,00365
ENSG00000105357	MYH14	7,784794	1,5E-05
ENSG00000105409	ATP1A3	2,185679	0,02406
ENSG00000105426	PTPRS	3,068414	1,6E-06
ENSG00000106415	GLCCI1	2,155459	0,00908

ENSG00000106785	TRIM14	2,084922	3E-129
ENSG00000107518	ATRNL1	2,871375	0,0019
ENSG00000107731	UNC5B	2,247625	0,00365
ENSG00000108342	CSF3	6,575165	0,00183
ENSG00000108387	SEPTIN4	3,10492	0,01557
ENSG00000108771	DHX58	2,871525	1,1E-47
ENSG00000108813	DLX4	2,14865	0,00316
ENSG00000108830	RND2	2,126616	0,00349
ENSG00000110446	SLC15A3	2,749993	2E-67
ENSG00000110848	CD69	5,238451	9,6E-10
ENSG00000111331	OAS3	2,66751	0
ENSG00000111335	OAS2	6,95967	0
ENSG00000112299	VNN1	3,359155	8E-304
ENSG00000112303	VNN2	2,842761	3,8E-10
ENSG00000115009	CCL20	6,967818	7,9E-52
ENSG00000115267	IFIH1	2,299511	2E-130
ENSG00000115457	IGFBP2	2,824974	0,0152
ENSG00000116962	NID1	8,673281	2,7E-07
ENSG00000117707	PROX1	2,275951	0,01228
ENSG00000118777	ABCG2	2,299981	0,00179
ENSG00000119508	NR4A3	2,382107	0,02512
ENSG00000119698	PPP4R4	3,252372	5,8E-05
ENSG00000119917	IFIT3	2,518329	2E-230
ENSG00000120337	TNFSF18	3,287529	0,00057
ENSG00000121753	ADGRB2	2,186788	0,04469
ENSG00000121966	CXCR4	2,025787	5,2E-22
ENSG00000123358	NR4A1	2,476096	6,4E-17
ENSG00000123610	TNFAIP6	2,114974	0,02005
ENSG00000124102	PI3	4,386429	3,8E-26
ENSG00000124875	CXCL6	4,411193	2E-252
ENSG00000125207	PIWIL1	4,673021	0,00294
ENSG00000125538	IL1B	2,487401	6,9E-66
ENSG00000125730	C3	2,708087	0
ENSG00000126259	KIRREL2	3,568157	0,00928
ENSG00000126709	IFI6	6,220685	0
ENSG00000130054	FAM155B	4,102929	0,03396
ENSG00000131409	LRRC4B	3,500818	0,03587
ENSG00000131459	GFPT2	3,01951	0,00116
ENSG00000132205	EMILIN2	2,036429	0,00541
ENSG00000132530	XAF1	4,318313	5E-214
ENSG00000132554	RGS22	5,96736	0,01289
ENSG00000132965	ALOX5AP	2,110418	1,5E-05
ENSG00000133106	EPSTI1	2,031717	4E-103
ENSG00000133328	PLAAT2	3,40192	0,04839
ENSG00000134258	VTCN1	2,05527	0,0006
ENSG00000134321	RSAD2	10,42191	8,1E-55
ENSG00000134326	CMPK2	7,657802	5E-107

ENSG00000134339	SAA2	2,535315	0
ENSG00000135063	FAM189A2	2,085335	9E-05
ENSG00000135114	OASL	5,488676	4E-173
ENSG00000135127	BICDL1	2,652351	0,00112
ENSG00000136244	IL6	2,094989	6E-187
ENSG00000136514	RTP4	2,361779	1,5E-14
ENSG00000136689	IL1RN	3,752394	0,00076
ENSG00000137273	FOXF2	6,03428	0,01098
ENSG00000137628	DDX60	2,215439	2E-203
ENSG00000137699	TRIM29	2,174281	0,00851
ENSG00000137745	MMP13	3,346591	8,6E-07
ENSG00000137878	GCOM1	3,180898	0,00777
ENSG00000137959	IFI44L	6,134191	0
ENSG00000137965	IFI44	4,407979	0
ENSG00000138622	HCN4	4,583993	0,0065
ENSG00000138642	HERC6	2,411545	8E-247
ENSG00000138646	HERC5	4,087495	3E-250
ENSG00000138670	RASGEF1B	2,054753	0,0331
ENSG00000139219	COL2A1	5,575815	0,03743
ENSG00000140379	BCL2A1	2,393147	2,6E-07
ENSG00000140465	CYP1A1	2,068735	0,01394
ENSG00000140832	MARVELD3	3,190149	0,00029
ENSG00000140968	IRF8	2,367371	0,04426
ENSG00000142224	IL19	2,522918	9,5E-05
ENSG00000143028	SYPL2	4,000048	0,04127
ENSG00000143603	KCNN3	2,619214	0,00049
ENSG00000144868	TMEM108	3,066745	1,2E-07
ENSG00000147003	CLTRN	2,672649	3,6E-08
ENSG00000148346	LCN2	6,537366	2,5E-58
ENSG00000148965	SAA4	3,112549	0,00015
ENSG00000152689	RASGRP3	2,356642	2,9E-05
ENSG00000154451	GBP5	6,897636	0,00063
ENSG00000155629	PIK3AP1	2,633508	1,8E-06
ENSG00000155961	RAB39B	5,745014	0,02335
ENSG00000156966	B3GNT7	2,800276	4E-101
ENSG00000157601	MX1	7,249281	0
ENSG00000158014	SLC30A2	5,716397	0,003
ENSG00000159403	C1R	2,610392	2,1E-84
ENSG00000160181	TFF2	5,575815	0,03743
ENSG00000160223	ICOSLG	3,578064	7,3E-44
ENSG00000161133	USP41	3,87173	2E-05
ENSG00000161270	NPHS1	5,575628	0,03634
ENSG00000162444	RBP7	5,887551	1,5E-08
ENSG00000162654	GBP4	2,431258	7,3E-23
ENSG00000162896	PIGR	3,937007	1,4E-34
ENSG00000162897	FCAMR	4,309981	2,4E-31
ENSG00000163121	NEURL3	3,124642	3,6E-19

ENSG00000163734	CXCL3	2,880464	8,9E-87
ENSG00000163739	CXCL1	2,618477	0
ENSG00000163874	ZC3H12A	2,214561	1E-110
ENSG00000164379	FOXQ1	3,379026	1,5E-63
ENSG00000164400	CSF2	2,829776	1E-164
ENSG00000165905	LARGE2	3,213456	0,01479
ENSG00000165949	IFI27	10,92809	2E-209
ENSG00000166313	APBB1	2,967075	1,2E-05
ENSG00000166523	CLEC4E	2,017878	4,9E-85
ENSG00000166670	MMP10	5,662277	0,02979
ENSG00000166831	RBPMS2	2,223747	0,01482
ENSG00000166920	C15orf48	4,204266	2,1E-17
ENSG00000167157	PRRX2	2,96035	1,4E-07
ENSG00000167244	IGF2	5,31348	3,1E-10
ENSG00000168062	BATF2	2,779494	1,8E-39
ENSG00000168427	KLHL30	2,549058	1,5E-11
ENSG00000168961	LGALS9	3,486424	0
ENSG00000169245	CXCL10	6,822872	0,00078
ENSG00000169248	CXCL11	7,108118	1,9E-05
ENSG00000169427	KCNK9	2,149048	0,02066
ENSG00000169429	CXCL8	2,956751	0
ENSG00000170011	MYRIP	4,145647	0,01915
ENSG00000170099	SERPINA6	2,764602	5,8E-47
ENSG00000170577	SIX2	4,075621	0,00053
ENSG00000171385	KCND3	4,854123	5,4E-05
ENSG00000171711	DEFB4A	7,597742	3,4E-05
ENSG00000171714	ANO5	2,006504	0,00647
ENSG00000172183	ISG20	3,430484	3,5E-97
ENSG00000172575	RASGRP1	3,41738	4,2E-10
ENSG00000173110	HSPA6	2,969648	3,9E-06
ENSG00000173432	SAA1	2,140363	0
ENSG00000173805	HAP1	2,794195	0,00737
ENSG00000173868	PHOSPHO1	3,021161	0,0073
ENSG00000173918	C1QTNF1	2,095842	6,9E-12
ENSG00000173926	MARCHF3	2,355851	0,00257
ENSG00000174672	BRSK2	2,616706	0,00534
ENSG00000175040	CHST2	2,069151	0,02854
ENSG00000175274	TP53I11	5,604765	0,00536
ENSG00000175352	NRIP3	3,088953	3,1E-05
ENSG00000175676	GOLGA8DP	2,856905	0,00251
ENSG00000176136	MC5R	8,619744	3,5E-07
ENSG00000176490	DIRAS1	4,372473	6,8E-05
ENSG00000176771	NCKAP5	2,66674	0,00048
ENSG00000176928	GCNT4	2,061762	0,00378
ENSG00000177181	RIMKLA	2,480499	0,02459
ENSG00000179046	TRIML2	2,435905	5,8E-10
ENSG00000179292	TMEM151A	5,662805	0,02879

ENSG00000182326	C1S	3,295052	4,7E-49
ENSG00000182580	EPHB3	5,065241	0,01789
ENSG00000182759	MAFA	5,745512	0,02373
ENSG00000183486	MX2	12,56161	2,7E-16
ENSG00000183785	TUBA8	3,197255	4,8E-05
ENSG00000184012	TMPRSS2	3,591826	1,9E-06
ENSG00000184489	PTP4A3	3,143964	0,01913
ENSG00000184792	OSBP2	2,726207	0,01062
ENSG00000184979	USP18	3,445343	2E-226
ENSG00000185507	IRF7	2,693012	1,6E-30
ENSG00000185519	FAM131C	2,730392	0,00738
ENSG00000185745	IFIT1	3,918607	1E-107
ENSG00000185818	NAT8L	3,151919	5,8E-08
ENSG00000185885	IFITM1	3,112794	0
ENSG00000187094	CCK	6,529117	0,00224
ENSG00000187608	ISG15	4,526517	6,8E-54
ENSG00000188505	NCCRP1	4,513582	0,00831
ENSG00000196611	MMP1	2,40203	7,8E-06
ENSG00000196684	HSH2D	4,401607	8,6E-57
ENSG00000196814	MVB12B	2,06128	0,04911
ENSG00000198780	FAM169A	4,051589	0,02574
ENSG00000198794	SCAMP5	2,600971	4,2E-07
ENSG00000198848	CES1	4,558061	2,2E-18
ENSG00000204642	HLA-F	2,039731	3,4E-66
ENSG00000205413	SAMD9	2,146964	6E-113
ENSG00000205436	EXOC3L4	3,009211	0,01577
ENSG00000206337	HCP5	2,138577	1,7E-72
ENSG00000206585	RNVU1-7	6,034004	0,01188
ENSG00000214548	MEG3	4,54797	0,00686
ENSG00000215182	MUC5AC	2,216969	0,00597
ENSG00000220793	RPL21P119	-5,57961	0,03192
ENSG00000225492	GBP1P1	5,121278	0,01693
ENSG00000228695	CES1P1	2,299926	7,3E-06
ENSG00000235385	LINC02154	3,247136	0,03377
ENSG00000236393	AC091806.1	2,195361	0,02016
ENSG00000237330	RNF223	3,465849	0,00387
ENSG00000243649	CFB	2,274668	6,4E-73
ENSG00000254887	AC010247.1	6,27639	0,00579
ENSG00000255071	SAA2-SAA4	3,161401	3,7E-24
ENSG00000256349	AP002748.5	-6,80652	0,00068
ENSG00000258791	LINC00520	2,208081	0,00192
ENSG00000258984	UBE2F-SCLY	-3,19367	0,03192
ENSG00000259342	AC025580.1	3,756926	0,00314
ENSG00000259354	AC025580.2	3,452455	0,04181
ENSG00000267261	AC099811.2	6,098231	0,00952
ENSG00000268089	GABRQ	6,219532	0,00624
ENSG00000268621	IGFL2-AS1	2,206933	9,6E-05

ENSG00000269547	AC011455.2	6,48185	0,0026
ENSG00000271503	CCL5	7,098496	0,00028
ENSG00000273590	SMIM11B	-6,32977	0,03199
ENSG00000276980	AC008760.2	2,056573	0,03603
ENSG00000277027	RMRP	-2,01199	0,03284
ENSG00000277117	FP565260.3	3,190958	2,4E-26
ENSG00000280236	OR12D2	6,432878	0,0031
ENSG00000283900	TPTEP2-CSNK1E	-5,85598	0,01502
ENSG00000284741	PDE11A	5,574309	0,04187
ENSG00000285162	AC004593.2	-6,3349	0,00341
ENSG00000285413	AP001056.2	5,745346	0,02335
ENSG00000285565	AL671762.1	2,252661	0,00256
ENSG00000285722	AC207130.1	5,823261	0,01897
ENSG00000285889	AL355312.5	-6,01064	0,00973
ENSG00000286088	AC073585.1	-6,59371	0,00153
ENSG00000286185	AC242842.3	5,66263	0,02879
ENSG00000287856	AL445524.2	6,529019	0,00231
ENSG00000288602	C8orf44-SGK3	-6,47787	0,00208
