# Supplementary material for: Human cytomegalovirus infection triggers a paracrine senescence loop in renal epithelial cells
Source: Commun Biol. 2024 Mar 8;7:292. doi: 10.1038/s42003-024-05957-5 (PMC10924099; doi:10.1038/s42003-024-05957-5)
Supplement: Supplementary file 2 — Description of Additional Supplementary Files [file 42003_2024_5957_MOESM2_ESM.pdf]

Description of Additional Supplementary Files

File name: Supplementary Data 1

Description: Source data behind the graphs in the manuscript.
