# Supplementary material for: Human cytomegalovirus infection triggers a paracrine senescence loop in renal epithelial cells
Source: Commun Biol. 2024 Mar 8;7:292. doi: 10.1038/s42003-024-05957-5 (PMC10924099; doi:10.1038/s42003-024-05957-5)
Supplement: Supplementary file 4 — Reporting summary [file 42003_2024_5957_MOESM4_ESM.pdf]

nature portfolio

Corresponding author(s): Marco De Andrea

Last updated by author(s): 2024/29/01

# Reporting Summary

Nature Portfolio wishes to improve the reproducibility of the work that we publish. This form provides structure for consistency and transparency in reporting. For further information on Nature Portfolio policies, see our Editorial Policies and the Editorial Policy Checklist.

## Statistics

For all statistical analyses, confirm that the following items are present in the figure legend, table legend, main text, or Methods section.

| n/a | Confirmed | |
|---|---|---|
| ☐ | ☒ | The exact sample size (*n*) for each experimental group/condition, given as a discrete number and unit of measurement |
| ☐ | ☒ | A statement on whether measurements were taken from distinct samples or whether the same sample was measured repeatedly |
| ☐ | ☒ | The statistical test(s) used AND whether they are one- or two-sided<br>*Only common tests should be described solely by name; describe more complex techniques in the Methods section.* |
| ☒ | ☐ | A description of all covariates tested |
| ☐ | ☒ | A description of any assumptions or corrections, such as tests of normality and adjustment for multiple comparisons |
| ☐ | ☒ | A full description of the statistical parameters including central tendency (e.g. means) or other basic estimates (e.g. regression coefficient) AND variation (e.g. standard deviation) or associated estimates of uncertainty (e.g. confidence intervals) |
| ☐ | ☒ | For null hypothesis testing, the test statistic (e.g. *F*, *t*, *r*) with confidence intervals, effect sizes, degrees of freedom and *P* value noted<br>*Give P values as exact values whenever suitable.* |
| ☒ | ☐ | For Bayesian analysis, information on the choice of priors and Markov chain Monte Carlo settings |
| ☒ | ☐ | For hierarchical and complex designs, identification of the appropriate level for tests and full reporting of outcomes |
| ☒ | ☐ | Estimates of effect sizes (e.g. Cohen's *d*, Pearson's *r*), indicating how they were calculated |

*Our web collection on statistics for biologists contains articles on many of the points above.*

## Software and code

Policy information about availability of computer code

| | |
|---|---|
| Data collection | Microsoft Exel 2016, ChemiDoc MP imaging system (BioRad), Real time CFX96 C1000 TOUCH thermal cycler (Bio-Rad Laboratories). All type of software used for transcriptomic data are described in the paper. Cytation 5 Cell Imaging Multi-Mode Reader (Agilent). Bio-PlexTM 200 System (Bio-Rad Laboratories). LAS X software (Leica Microsystems). |
| Data analysis | GraphPad 9.20, Image Lab 6.1. STAR program was used to align reads to the reference human hg38 genome. All type of analysis used for transcriptomic data are described in the paper. LAS X software (Leica Microsystems). Bio-Plex Manager Software (Bio-Rad Laboratories). BioTek Gen5 Software for imaging & Microscopy was used for the Cytation 5 Cell Imaging Multi-Mode Reader (Agilent). Real-time data were evaluated with CFX Maestro Software (Bio-Rad Laboratories). |

For manuscripts utilizing custom algorithms or software that are central to the research but not yet described in published literature, software must be made available to editors and reviewers. We strongly encourage code deposition in a community repository (e.g. GitHub). See the Nature Portfolio guidelines for submitting code & software for further information.

## Data

Policy information about availability of data

All manuscripts must include a data availability statement. This statement should provide the following information, where applicable:

- Accession codes, unique identifiers, or web links for publicly available datasets
- A description of any restrictions on data availability
- For clinical datasets or third party data, please ensure that the statement adheres to our policy

*Provide your data availability statement here.*

## Research involving human participants, their data, or biological material

Policy information about studies with human participants or human data. See also policy information about sex, gender (identity/presentation), and sexual orientation and race, ethnicity and racism.

| | |
|---|---|
| Reporting on sex and gender | *Use the terms sex (biological attribute) and gender (shaped by social and cultural circumstances) carefully in order to avoid confusing both terms. Indicate if findings apply to only one sex or gender; describe whether sex and gender were considered in study design; whether sex and/or gender was determined based on self-reporting or assigned and methods used. Provide in the source data disaggregated sex and gender data, where this information has been collected, and if consent has been obtained for sharing of individual-level data; provide overall numbers in this Reporting Summary. Please state if this information has not been collected. Report sex- and gender-based analyses where performed, justify reasons for lack of sex- and gender-based analysis.* |
| Reporting on race, ethnicity, or other socially relevant groupings | *Please specify the socially constructed or socially relevant categorization variable(s) used in your manuscript and explain why they were used. Please note that such variables should not be used as proxies for other socially constructed/relevant variables (for example, race or ethnicity should not be used as a proxy for socioeconomic status). Provide clear definitions of the relevant terms used, how they were provided (by the participants/respondents, the researchers, or third parties), and the method(s) used to classify people into the different categories (e.g. self-report, census or administrative data, social media data, etc.) Please provide details about how you controlled for confounding variables in your analyses.* |
| Population characteristics | *Describe the covariate-relevant population characteristics of the human research participants (e.g. age, genotypic information, past and current diagnosis and treatment categories). If you filled out the behavioural & social sciences study design questions and have nothing to add here, write "See above."* |
| Recruitment | *Describe how participants were recruited. Outline any potential self-selection bias or other biases that may be present and how these are likely to impact results.* |
| Ethics oversight | *Identify the organization(s) that approved the study protocol.* |

Note that full information on the approval of the study protocol must also be provided in the manuscript.

# Field-specific reporting

Please select the one below that is the best fit for your research. If you are not sure, read the appropriate sections before making your selection.

☒ Life sciences ☐ Behavioural & social sciences ☐ Ecological, evolutionary & environmental sciences

For a reference copy of the document with all sections, see nature.com/documents/nr-reporting-summary-flat.pdf

# Life sciences study design

All studies must disclose on these points even when the disclosure is negative.

| | |
|---|---|
| Sample size | The sample size used in this study was determined based on the expense data colletion, and the need to have sufficient statistical power. As described in the paper, the sample sizes used were demeed appropriate as n=3. In Transcriptomic analysis n=2 |
| Data exclusions | Data were generally not excluded from the analyses |
| Replication | All data presented were obtained from three or two independent experiment with similar outcomes (see figure legends) |
| Randomization | Randomization was not used. |
| Blinding | Data collection and analysis were not performed blind to the condition of the experiments, because experiments were performed and analyzed by the same researchers. However, all experiments and analysis were performed objective and unbiased. |

# Reporting for specific materials, systems and methods

We require information from authors about some types of materials, experimental systems and methods used in many studies. Here, indicate whether each material, system or method listed is relevant to your study. If you are not sure if a list item applies to your research, read the appropriate section before selecting a response.

## Materials & experimental systems

| n/a | Involved in the study |
|---|---|
| ☐ | ☒ Antibodies |
| ☐ | ☒ Eukaryotic cell lines |
| ☐ | ☐ Palaeontology and archaeology |
| ☒ | ☐ Animals and other organisms |
| ☒ | ☐ Clinical data |
| ☒ | ☐ Dual use research of concern |
| ☒ | ☐ Plants |

## Methods

| n/a | Involved in the study |
|---|---|
| ☒ | ☐ ChIP-seq |
| ☒ | ☐ Flow cytometry |
| ☒ | ☐ MRI-based neuroimaging |

## Antibodies

Antibodies used

Monoclonal anti-HCMV IEA (8P1215, Virusys )
In house-produced rabbit polyclonal anti-IEA (DOI: 10.3390/v10100567)
Monoclonal anti-HCMV pp28 (P1207, Virusys)
Monoclonal anti-HCMV UL44 (P1202, Virusys)
Goat anti-mouse IgG-Alexa Fluor 488 (A11001, Thermo Fisher Scientific)
Goat anti-mouse IgG-Alexa Fluor 568 (A-11036, Thermo Fisher Scientific)
Monoclonal NF-κB (L8F6 6956, Cell Signaling Technology)
Monoclonal γH2AX (JBW301, Sigma-Aldrich)
Monoclonal anti-p16INK4a (E6N8P) (18769S, Cell Signaling Technology),
Monoclonal anti-β-actin (A1978, Sigma-Aldrich)
Mouse IgG-HRP antibody (NA931V, GE Healthcare)
Rabbit IgG-HRP antibody (A6154, Sigma)
Anti-HCMV blend cocktail 8B1.2, 1G5.2 & 2D4.2 mouse monoclonal primary antibody, catalogue number 760-4

Validation

Validation was provided by the vendor for all antibodies used in this study. In house-produced rabbit polyclonal anti-IEA was validated by previous users (DOI: 10.3390/v10100567)

## Eukaryotic cell lines

Policy information about cell lines and Sex and Gender in Research

Cell line source(s)

ARPE-19 (ATCC)
RPTEC-hTERT (Evercyte)
HFF (ATCC)

Authentication

ARPE-19: ARPE-19 is a spontaneously arising retinal pigment epithelia (RPE) cell line derived in 1986 by Amy Aotaki-Keen from the normal eyes of a 19-year-old male who died from head trauma in a motor vehicle accident. Organism: Homo sapiens, human. Cell type: epithelial cell. Morphology: epithelial. Growth properties: Adherent.

RPTEC: RPTEC/TERT1 cells are characterized by the typical epithelial morphology and formation of domes when grown to high cell densities, which demonstrates active transport of water from the apical to the basolateral side. Moreover, the cells form continuous belts of E-cadherin and ZO-1 at cell-cell contacts. Organism: homo sapiens. Tissue, cell type: kidney cortex, male donor. Morphology: epithelial, cobblestone morphology. Life span extension: ectopic expression of hTERT.

HFF: The cell line was established by ATCC in 2003 from normal human foreskin pooled from two individuals. Age: neonate. Gender: Male. Organism: Homo sapiens, human. Cell type: fibroblast. Morphology: fibroblast. Tissue: Skin; Foreskin.

Mycoplasma contamination

We confirm that all cell lines tested negative for mycoplasma contamination

Commonly misidentified lines
(See ICLAC register)

No commonly misidentified cell lines were used in the study

## Palaeontology and Archaeology

Specimen provenance

*Provide provenance information for specimens and describe permits that were obtained for the work (including the name of the issuing authority, the date of issue, and any identifying information). Permits should encompass collection and, where applicable, export.*

| | |
|---|---|
| Specimen deposition | *Indicate where the specimens have been deposited to permit free access by other researchers.* |
| Dating methods | *If new dates are provided, describe how they were obtained (e.g. collection, storage, sample pretreatment and measurement), where they were obtained (i.e. lab name), the calibration program and the protocol for quality assurance OR state that no new dates are provided.* |

☐ Tick this box to confirm that the raw and calibrated dates are available in the paper or in Supplementary Information.

| | |
|---|---|
| Ethics oversight | *Identify the organization(s) that approved or provided guidance on the study protocol, OR state that no ethical approval or guidance was required and explain why not.* |

Note that full information on the approval of the study protocol must also be provided in the manuscript.

## Plants

| | |
|---|---|
| Seed stocks | *Report on the source of all seed stocks or other plant material used. If applicable, state the seed stock centre and catalogue number. If plant specimens were collected from the field, describe the collection location, date and sampling procedures.* |
| Novel plant genotypes | *Describe the methods by which all novel plant genotypes were produced. This includes those generated by transgenic approaches, gene editing, chemical/radiation-based mutagenesis and hybridization. For transgenic lines, describe the transformation method, the number of independent lines analyzed and the generation upon which experiments were performed. For gene-edited lines, describe the editor used, the endogenous sequence targeted for editing, the targeting guide RNA sequence (if applicable) and how the editor was applied.* |
| Authentication | *Describe any authentication procedures for each seed stock used or novel genotype generated. Describe any experiments used to assess the effect of a mutation and, where applicable, how potential secondary effects (e.g. second site T-DNA insertions, mosiacism, off-target gene editing) were examined.* |
